# Decoding Choroid Plexus Pathology in Alzheimer's Disease: A Longitudinal Radiomics Approach for Prodromal Identification and Risk Stratification

**DOI:** 10.1002/cns.70987

**Published:** 2026-06-15

**Authors:** Feiyue Yin, Xiaohua Wang, Xiao Chen, Jinju Sun, Kai Zhang, Xiaojuan Dong, Wenwen Wang, Peng Zeng, Binglan Li, Lisha Nie, Dan Luo, Yongmei Li, Tianyou Luo, Alzheimer's Disease Neuroimaging Initiative

**Affiliations:** ^1^ Department of Radiology The First Affiliated Hospital of Chongqing Medical University Chongqing China; ^2^ Department of Radiology, Sichuan Provincial People's Hospital University of Electronic Science and Technology of China Chengdu China; ^3^ Department of Nuclear Medicine Daping Hospital of Army Medical University Chongqing China; ^4^ GE Healthcare MR Research China Beijing China

**Keywords:** Alzheimer's disease, choroid plexus, cognitive impairment, machine learning, radiomics

## Abstract

**Aims:**

Alzheimer's disease (AD) is the most prevalent neurodegenerative disorder, and its identification of its prodromal stages remains a challenge. The choroid plexus (CP) plays a crucial role in AD pathology. This study aims to develop the CP radiomics model to distinguish AD from mild cognitive impairment (MCI) patients and predict the risk of MCI progression to AD.

**Methods:**

Radiomics features of CP were derived from magnetic resonance imaging (MRI) data, utilizing scans derived from the Alzheimer's Disease Neuroimaging Initiative (ADNI) cohort and a local institutional cohort. A range of 12 classic machine learning algorithms was utilized for model construction. Additionally, we assessed the partial correlations of CP radiomics features with the Mini‐Mental State Examination (MMSE) score and with CSF biomarkers.

**Results:**

In the MCI versus AD classification task, the CP model attained a best AUC of 0.794. This performance was further boosted to an AUC of 0.907 upon integration with clinical features. In the model predicting MCI‐to‐AD conversion, the CP model achieved an AUC of 0.745, which increased to 0.908 after incorporating clinical features. Furthermore, the high‐risk group, as defined by the radiomics model, had a significantly shorter time to AD conversion (HR = 2.201, *p* < 0.001). CP radiomics features showed significant correlations with MMSE and CSF biomarkers (*p* < 0.05).

**Conclusion:**

The machine learning model based on CP radiomics features effectively differentiates AD from MCI patients and predicts the risk of MCI progression to AD, offering new insights into the role of the CP in AD pathophysiology.

## Graphical abstract

A machine learning model utilizing choroid plexus (CP) radiomics effectively distinguishes Alzheimer's disease (AD) from mild cognitive impairment (MCI) and predicts MCI‐to‐AD progression risk. After integration with clinical features, the model exhibits strong predictive performance. The CP radiomics features correlate significantly with cognitive and cerebrospinal fluid biomarkers, highlighting the CP role in AD pathology and offering a potential tool for early diagnosis.

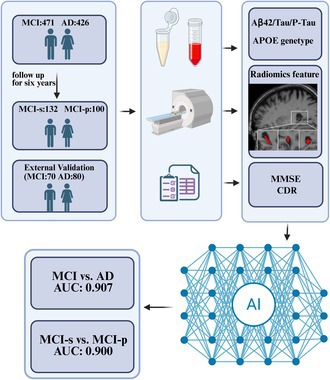

## 1 Introduction

Alzheimer's disease (AD), the most prevalent neurodegenerative disorder and primary cause of dementia, is pathologically defined by the accumulation of amyloid‐beta (Aβ) plaques and neurofibrillary tau tangles, processes that typically commence decades prior to clinical symptom onset [1]. The high incidence and severe consequences of AD make it a heavy burden on both the healthcare system and society [2]. Mild cognitive impairment (MCI) is an important precursor stage for the development of AD, with MCI patients having a 10%–15% risk of progressing to AD each year [3]. In developing countries, the prevalence of MCI can reach as high as 16% [4]. Therefore, timely detection and intervention are crucial for preventing the onset of AD. However, the diagnosis of AD, especially accurate identification at the prodromal stage, still faces many challenges. In clinical practice, the in vivo diagnostic workup depends on biomarkers detected via positron emission tomography (PET) scans and cerebrospinal fluid (CSF) examinations. Nevertheless, the high cost of PET and the invasive nature of CSF collection limit their widespread use in primary care settings [5, 6]. Moreover, accurately predicting the progression from MCI to AD remains a critical unmet need in clinical management. Accordingly, there is an urgent need for further research and development of more reliable and cost‐effective diagnostic methods to improve the identification of prodromal AD, and the prognostic prediction of AD conversion in individuals with MCI.

The choroid plexus (CP) is central to the pathogenesis of AD. Growing evidence indicates that ineffective protein clearance, as opposed to protein overproduction, constitutes the principal driver of AD progression [3, 7]. Most of the proposed clearing mechanisms are related to CSF, including protein degradation and cellular uptake, transport across the blood–brain barrier (BBB) and blood‐CSF barrier, interstitial fluid volume flow, and absorption of CSF into the circulatory and lymphatic systems [8]. The CP serves multiple physiological roles, including CSF production and the maintenance of the blood‐CSF barrier, which is established by tight junctions connecting choroidal epithelial cells [9]. The CP functions as a vital source of nutrients, a clearance pathway, and a site of immune surveillance. It features a richly vascularized stromal core with fenestrated capillaries, overlaid by epithelial cells that are interlinked via tight junctions [9, 10]. Studies indicate that structural and functional changes in the CP are already present at early pathological stages of AD [11]. For example, under stress conditions, increased BBB permeability may allow harmful substances to enter the brain, triggering neuroinflammation and neuronal damage [12, 13]. Therefore, the CP may become a potential source of novel biomarkers for AD.

Traditional structural MRI, such as hippocampal volume, has certain limitations in the diagnosis of MCI and AD. These changes typically manifest in the later stages of the disease [14], resulting in insufficient sensitivity for the identification of prodromal AD detection and difficulty in capturing subtle pathophysiological changes. Functional imaging techniques like Aβ‐PET and Tau‐PET offer higher specificity, but their high costs, radiation exposure, and limited accessibility remain major constraints [15]. Radiomics, which involves the high‐throughput extraction of numerous quantitative features from medical images, can transform images into high‐dimensional, data‐rich information. This approach reveals image information that is difficult to detect with the naked eye, reflecting the microstructural and functional heterogeneity of tissues, and provides non‐invasive, quantitative, and comprehensive biomarkers [16]. Research on radiomics of CP, a critical target, holds promise for capturing complex pathological changes associated with MCI and AD at earlier and more sensitive stages. Currently, radiomics research in neuroscience primarily focuses on well‐established structures like the hippocampus, cortex, and white matter. An emerging line of radiomics research has begun to focus on the cerebellum along the AD continuum. Several studies have shown that cerebellum‐derived radiomics signatures and network features based on T1, T2, and fluid‐attenuated inversion recovery (FLAIR) sequences can effectively distinguish individuals with MCI from cognitively normal controls and contribute to AD diagnosis and risk stratification [17, 18, 19, 20]. These findings collectively suggest that cerebellar radiomics features hold promise as novel biomarkers for the early identification and progression prediction of AD [21]. In contrast, the CP remains a relatively under‐explored target, despite its emerging critical role [22]. To date, there is a lack of systematic studies investigating both AD and MCI classification, as well as the prediction of MCI progression to AD based on CP radiomics features. Therefore, comprehensive exploration of CP radiomics characteristics holds significant clinical and research value.

Hence, this study aims to develop and validate a machine learning model based on CP radiomics features to (1) differentiate AD from MCI and (2) predict the risk of MCI progression to AD. (3) Additionally, we analyzed the correlation between CP radiomics features and clinical characteristics to elucidate the role of CP within the AD pathology.

## 2 Methods

### 2.1 Participants

**FIGURE 1:**
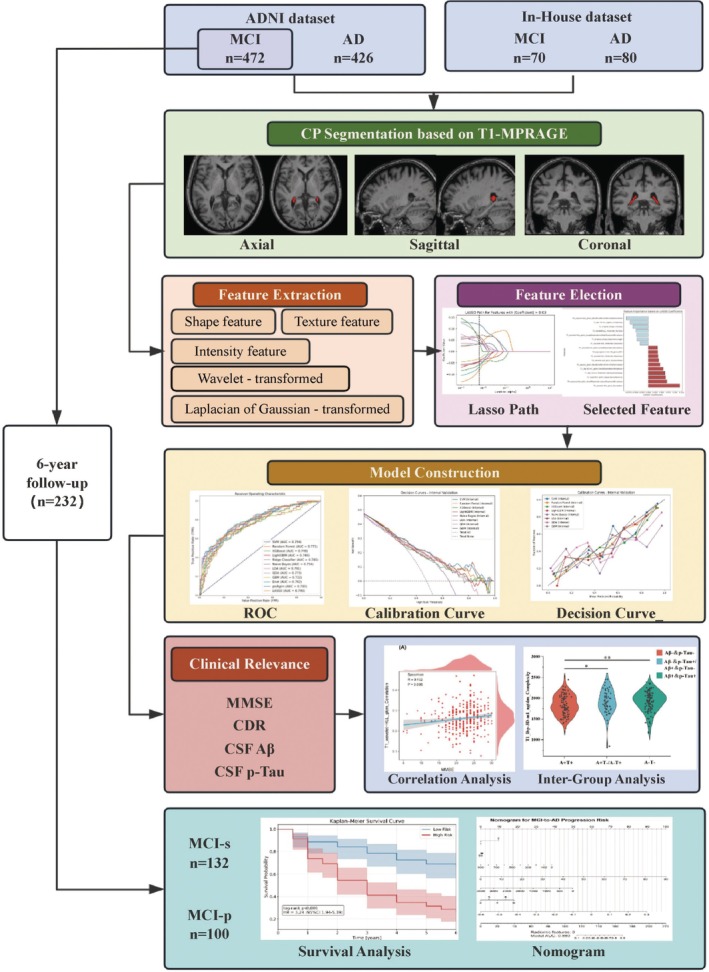
Flowchart of the data analysis pipeline. AD, Alzheimer's disease; ADNI, Alzheimer's Disease Neuroimaging Initiative; Aβ, amyloid‐beta; CDR, Clinical Dementia Rating; CP, choroid plexus; CSF, cerebrospinal fluid; MCI, mild cognitive impairment; MCI‐p, MCI‐progression; MCI‐s, MCI‐stable; MMSE, Mini‐Mental State Examination; p‐Tau, phosphorylated tau; ROC, receiver operating characteristic; T1‐MPRAGE, T1 weighted magnetization‐prepared rapid gradient echo.

This study included a primary cohort of 898 participants (472 MCI, 426 AD) from the ADNI database and an independent external validation cohort of 150 subjects (70 MCI, 80 AD) from Daping Hospital of Army Medical University. Among MCI subjects with longitudinal follow‐up (*n* = 232), 100 progressed to AD and 132 remained stable (Figure 1). Detailed inclusion criteria, diagnostic definitions, and biomarker data are provided in Method S1.

### 2.2 3D‐T1 Weighted MRI Protocol

This study utilized standardized T1‐weighted anatomical imaging acquired through volumetric 3D magnetization‐prepared rapid gradient echo (MPRAGE) sequences or equivalent protocols with slightly different resolutions across patients. While slight variations in resolution existed across participants, all scans were obtained on a 3.0 T magnetic resonance scanner in the ADNI cohort. The complete imaging protocols are publicly available through the ADNI database (http://adni.loni.usc.edu/methods/documents/, accessed 19 October 2025). The in‐house 3D‐T1 weighted MRI data were collected on a 3.0 T scanner (Signa, GE Healthcare) equipped with a 12‐channel phased‐array head coil at Daping Hospital of Army Medical University. Detailed acquisition parameters are described in Method S1.

### 2.3 Choroid Plexus Segmentation and Feature Extraction

Data standardization began with N4 bias correction, which was applied to remove low‐frequency intensity non‐uniformities. Subsequently, *Z*‐score normalization standardized signal intensities. Finally, all images were resampled to a uniform 1 × 1 × 1 mm^3^ voxel size.

The CP segmentation was performed automatically on 3D T1‐MPRAGE images utilizing a previously established UX‐Net model [23]. The detailed methodology for segmentation is described in Method S2. All generated CP masks underwent manual review and minor correction to ensure segmentation quality. The segmentation performance of the 3D UX‐Net model was evaluated in a representative subset of 50 participants. This subset was strategically selected to include 40 subjects from the ADNI database (20 MCI and 20 AD) and 10 subjects from the local in‐house dataset (5 MCI and 5 AD) to ensure cross‐cohort generalizability. Ground truth manual annotations were independently performed on these 50 cases by a neuroradiologist (Y.F.Y., with 3 years of experience). Examples of CP segmentation by UX‐Net are shown in Figure S1. The automated segmentation achieved excellent agreement with the manual gold standard, yielding a mean Dice similarity coefficient of 0.8371, a Jaccard index of 0.7285, and a 95th percentile Hausdorff distance (HD95) of 1.68 (Table S1). Comparison between raw automated masks and manually refined masks (by D.X.J., with 7 years of experience) revealed negligible differences, with a mean Dice of 0.9702, Jaccard of 0.9426, and HD95 of 0.43, confirming that the automated segmentation already achieved high quality with minimal need for refinement (Table S2).

Following this, radiomics features were extracted from the original T1‐MPRAGE images based on the finalized CP masks. This extraction was performed using the FeAture Explorer (FAE, version v0.6.0.7z) software [24] (accessed 19 October 2025) in accordance with the Image Biomarker Standardization Initiative (IBSI) guidelines. Finally, we extracted a comprehensive set of 1688 radiomics features from the CP, encompassing first‐order, shape, and texture characteristics from both original and filtered images, all standardized via *Z*‐score normalization (Method S3).

### 2.4 Features Selection and Model Construction

A multi‐stage feature selection pipeline was applied to identify robust predictors, integrating statistical testing, correlation filtering, mutual information analysis, and least absolute shrinkage and selection operator (LASSO) regression (Method S4). The final number of selected features was determined in a data driven manner using the optimal regularization parameter (*λ*) in LASSO rather than being specified in advance, ensuring a balance between model complexity and predictive performance. Subsequently, multiple machine learning classifiers were trained using stratified cross‐validation with the Synthetic Minority Over‐sampling Technique (SMOTE) oversampling and evaluated on an independent test set via receiver operating characteristic (ROC) analysis (Method S5). SHapley Additive exPlanations (SHAP) were used to interpret model behavior and assess the impact of individual features on predictions (Method S6).

### 2.5 Prediction Performance Analysis of the CP Radiomics Models

To extract a comprehensive risk score (Radscore) from radiomics features, we employed LASSO regression for feature selection and determined the optimal regularization parameter *λ* through cross‐validation. Using the median Radscore value as the threshold, participants were stratified into high‐risk and low‐risk groups. Kaplan–Meier survival curves were plotted to demonstrate the cumulative probability of AD progression‐free survival, with between‐group differences in survival distributions compared using the log‐rank test.

To assess the predictive performance of CP radiomics models for MCI to AD progression risk stratification, we conducted Cox proportional hazards regression, which was adjusted for clinically relevant covariates including age, sex, and Apolipoprotein E (APOE) ε4 carrier status to control for potential confounding effects. Adjusted hazard ratios (HR) were calculated to quantify the increased risk for conversion to AD in the high‐risk group compared to the low‐risk group.

To translate the model into a clinically applicable tool, a nomogram incorporating both the Radscore and key clinical features was developed, and its improved performance over a clinical‐only model was rigorously validated through calibration curves, Net Reclassification Index (NRI), and Integrated Discrimination Improvement (IDI) (Method S7). Logistic regression was chosen for the final nomogram construction to ensure clinical interpretability through a transparent scoring system based on points derived from regression coefficients. While a Cox‐based nomogram could provide predictions of time to event, our primary aim was to estimate overall conversion risk within a standardized period of follow up, which is more directly applicable in clinical screening.

### 2.6 Statistical Analysis

Statistical analyses were conducted using SPSS software (version 27.0, IBM Corporation) and RStudio (version 2024.09.1). Categorical variables, such as sex and APOE ε4 carrier status, were expressed as percentages and compared between groups using the chi‐squared test. For continuous demographic and neuropsychological variables, normality was assessed with the Kolmogorov–Smirnov test. Normally distributed variables were summarized as mean (standard deviation), whereas non‐normally distributed data were expressed as median (interquartile range). Group comparisons were performed using independent samples *t*‐tests or Mann–Whitney *U* tests, as appropriate. Differences among groups were compared using a one‐way analysis of variance (ANOVA) and post hoc tests. Performance metrics were derived from ROC curve analysis. Spearman correlation coefficients were calculated between the key radiomics features and Mini‐Mental State Examination (MMSE) in AD and MCI subjects to assess the associations between the CP radiomics features and cognitive status with the adjustment of age, sex, and APOE ε4 status. Pearson correlations between CP radiomics features and CSF Aβ42 and p‐Tau levels were computed adjusting the *p* value using the Benjamini–Hochberg false‐discovery‐rate (FDR) procedure. A two‐tailed *p* < 0.05 was regarded as statistically significant. A heatmap was generated with the R studio; only correlations with FDR‐corrected *p* < 0.05 were marked with an asterisk.

## 3 Results

### 3.1 Demographic and Clinical Characteristics of the Datasets

The demographics and clinical characteristics of the participants included in this study are shown in Table 1. A total of 898 participants from the ADNI database were included in this study, comprising 472 patients with MCI and 426 patients with AD. Within the MCI cohort, 232 patients were followed up for 6 years. Of these, 132 MCI remained clinically stable (MCI‐s), while 100 MCI progressed to AD (MCI‐p). Due to a significant baseline age difference between the MCI‐s and MCI‐p groups (*p* = 0.007), propensity score matching (PSM) based on age and sex was performed. This resulted in a matched cohort of 71 MCI‐s and 71 MCI‐p for prediction model construction.

**TABLE 1:** Demographics and clinical characteristics of the participants included in this study.

Variables	AD vs. MCI classification			MCI‐s vs. MCI‐p			MCI‐s vs. MCI‐p after PSM		
	MCI (*n* = 472)	AD (*n* = 426)	*p*	MCI‐s (*n* = 132)	MCI‐p (*n* = 100)	*p*	MCI‐s (*n* = 71)	MCI‐p (*n* = 71)	*p*
Age, mean (SD), years	72.89 (7.21)	72.15 (7.41)	0.135	70.03 (6.44)	72.53 (7.51)	0.007*	71.45 (6.03)	71.57 (6.32)	0.903
Female, No. (%)	206 (43.6)	196 (46.0)	0.454	80 (60.6)	58 (58)	0.705	44 (61.9)	44 (61.9)	1.0
APOE ε4 status			< 0.001*			< 0.001*			< 0.001*
Non‐carriers, No. (%)	289 (61.2)	193 (45.3)		87 (66.4)	39 (39.0)		47 (66.2)	22 (31.0)	
ε4 carriers, No. (%)	162 (34.3)	232 (54.4)		44 (33.5)	60 (60.0)		24 (33.8)	49 (69.0)	
Tau, mean (SD), pg/mL	246.8 (185.6, 330.9)	332.5 (268.5, 446.7)	< 0.001*	227.0 (168.9, 281.8)	312.8 (240.8, 417.9)	< 0.001*	237.1 (194.5, 309.3)	291.7 (239.7, 414.6)	0.005*
p‐Tau, mean (SD), pg/mL	22.83 (16.30, 32.51)	33.06 (25.26, 43.54)	< 0.001*	20.02 (14.9, 25.8)	30.88 (22.3, 41.3)	< 0.001*	21.82 (17.4, 28.5)	29.6 (21.0, 40.8)	0.004*
Aβ42, mean (SD), pg/mL	917.3 (669.3, 1381.3)	617.5 (474.9, 772.6)	< 0.001*	1162.0 (839.5, 1689.0)	669.3 (533.6, 835.7)	< 0.001*	1119.5 (788.6, 1685.7)	655.2 (518.1, 823.6)	< 0.001*

*Note:* Values are expressed as mean (standard deviation) for normally distributed continuous variables and as median (interquartile range) for non‐normally distributed continuous variables. Categorical variables are presented as number (percentage). *p*‐values were calculated using the Student's *t*‐test or Mann–Whitney *U*‐test for continuous variables and the chi‐squared test or Fisher's exact test for categorical variables, as appropriate. Bold values and asterisks indicates *p*‐value< 0.05.

Abbreviations: Aβ, amyloid‐beta; AD, Alzheimer's disease; APOE, Apolipoprotein E; MCI, mild cognitive impairment; MCI‐p, MCI‐progression; MCI‐s, MCI‐stable; p‐Tau, phosphorylated tau; PSM, propensity score matching.

In the classification model distinguishing MCI and AD, no significant differences were observed in age (*p* = 0.135) or sex (*p* = 0.454). However, significant differences were found in several biomarkers: the AD cohort had a higher prevalence of APOE ε4 carriers (*p* < 0.001), higher CSF levels of Tau (*p* < 0.001) and p‐Tau (*p* < 0.001), but lower CSF levels of Aβ42 (*p* < 0.001) compared to the MCI cohort.

Prior to PSM in the MCI follow‐up cohort (*n* = 231), the MCI‐p group was significantly older than the MCI‐s group (*p* = 0.007), but sex distribution was similar (*p* = 0.705). The MCI‐p group also had a significantly higher prevalence of APOE ε4 carriers (*p* < 0.001), higher CSF levels of Tau (*p* < 0.001) and p‐Tau (*p* < 0.001), and lower CSF levels of Aβ42 protein (*p* < 0.001) compared to the MCI‐s group.

After PSM selection, the groups were well‐balanced for age (*p* = 0.903) and sex (*p* = 1.00). Despite this matching, the MCI‐p group retained significantly higher rates of APOE ε4 carriage (*p* < 0.001), higher CSF levels of Tau (*p* = 0.005) and p‐Tau (*p* = 0.004), and lower CSF levels of Aβ42 (*p* < 0.001) compared to the MCI‐s group.

### 3.2 Performance Evaluation of Classification and Prediction Model Construction

#### 3.2.1 Classification Model

After feature selection, 17 CP radiomics features were retained in discriminating AD from MCI using radiomics feature‐based models. Among the 17 key features, T1_original_shape_Flatness and T1_original_shape_MajorAxisLength belong to the shape category, suggesting a tendency toward a flatter morphology. First‐order intensity features, including T1_lbp‐3D‐m2_firstorder_InterquartileRange, T1_wavelet‐HLL_firstorder_Skewness, T1_wavelet‐LLL_firstorder_Kurtosis, and T1_wavelet‐HHL_firstorder_Skewness, characterize the distribution of voxel intensities within the CP, suggesting the presence of localized high‐intensity regions and asymmetric gray‐level distributions. The remaining features are texture‐based, capturing increased gray‐level heterogeneity and structural complexity of the CP. Overall, the CP of AD patients appears to exhibit a flatter shape, more heterogeneous gray‐level distribution, and increased texture complexity, which may serve as imaging characteristics associated with AD. To evaluate their importance, we utilized the SHAP framework to quantify the individual contribution of each feature to model discrimination, with the results presented in Figure S2.

**FIGURE 2:**
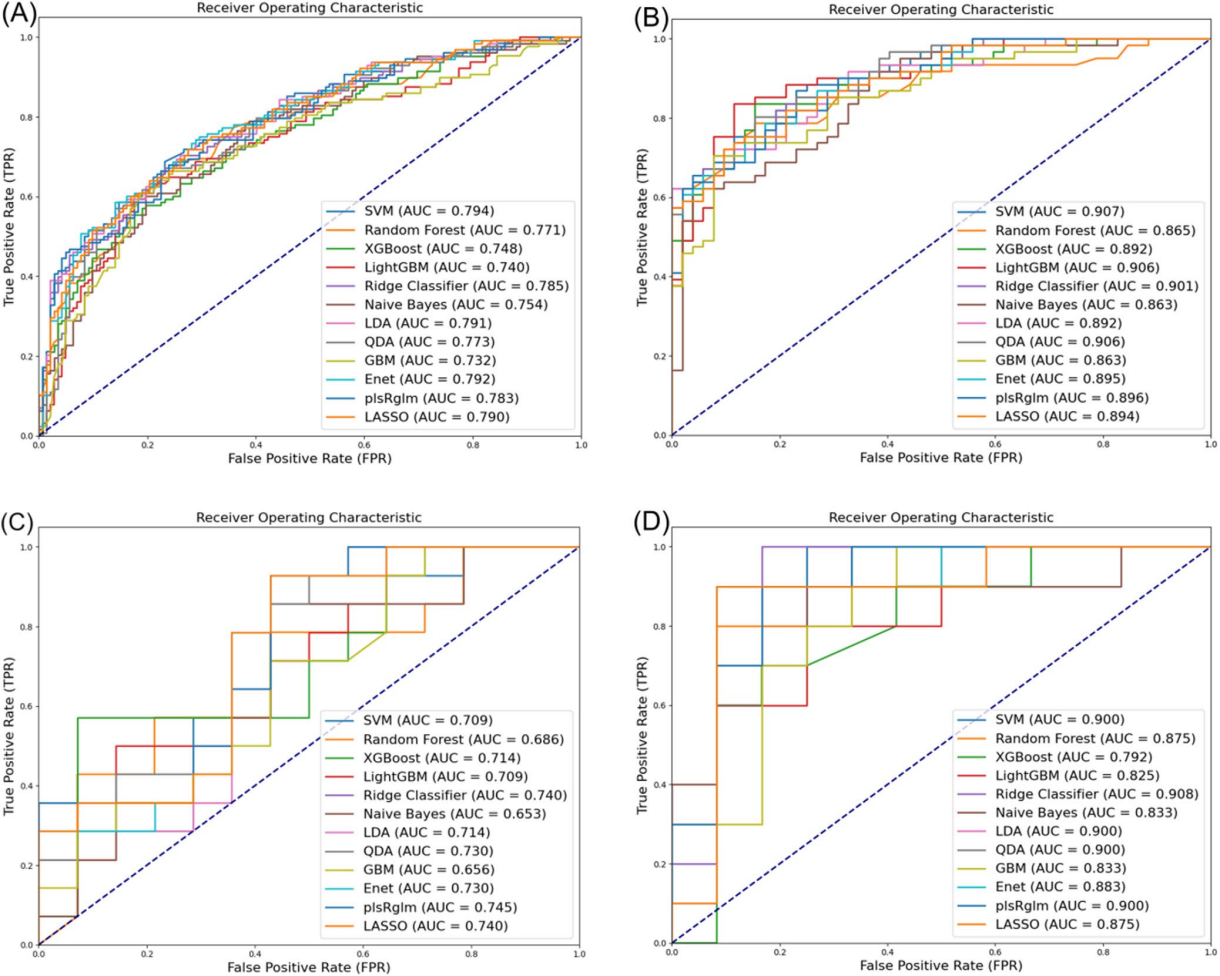
Performance of classification and prediction models based on CP radiomics features and clinical information. (A) ROC curves of radiomics models for differentiating AD and MCI. (B) ROC curves of combined clinical‐radiomics models for differentiating AD and MCI. (C) ROC curves of radiomics models for differentiating MCI‐s and MCI‐p. (D) ROC curves of combined clinical‐radiomics models for differentiating MCI‐s and MCI‐p. AD, Alzheimer's disease; CP, choroid plexus; Enet, Elastic Net; GBM, Gradient Boosting Machine; LASSO, Least Absolute Shrinkage and Selection Operator; LDA, Linear Discriminant Analysis; LightGBM, Light Gradient Boosting Machine; MCI, mild cognitive impairment; MCI‐p, MCI‐progression; MCI‐s, MCI‐stable; NB, Naive Bayes; plsRglm, Partial Least Squares Regression Generalized Linear Model; QDA, Quadratic Discriminant Analysis; RC, Ridge Classifier; RF, Random Forest; ROC, receiver operating characteristic; SVM, Support Vector Machine; XGBoost, eXtreme Gradient Boosting.

In discriminating AD from MCI using radiomics feature‐based models, several classifiers demonstrated robust performance: Support Vector Machine (SVM), Elastic Net (Enet), Linear Discriminant Analysis (LDA), LASSO, Ridge Classifier (RC), Partial Least Squares Regression Generalized Linear Model (plsRglm), Quadratic Discriminant Analysis (QDA), and Random Forest (RF) achieved area under the curve (AUC) ranging from 0.771 to 0.794. Among these, SVM yielded the highest AUC (0.794, 95% CI: 0.741–0.847), with precision, recall, and specificity of 0.727, 0.688, and 0.768, respectively. In addition, Light Gradient Boosting Machine (LightGBM), eXtreme Gradient Boosting (XGBoost), Naive Bayes (NB), and Gradient Boosting Machine (GBM) exhibited moderate performance (AUCs: 0.732–0.748) (Figure 2 and Table S3). In the external validation cohort. RF, XGBoost and LightGBM achieved high AUC values between 0.750 and 0.786. Here, the XGBoost performed best, achieving an AUC of 0.786 (95% CI: 0.586–0.929) with a precision of 0.769, recall of 0.714, and specificity of 0.800 as shown in Figure S3 and Table S4.

Integration of clinical features with radiomics features significantly enhanced classification efficacy. The SVM model emerged as optimal, achieving an AUC of 0.907 (95% CI: 0.848–0.953), with precision, recall, and specificity of 0.885, 0.754, and 0.885, respectively. Other high‐performing classifiers included LightGBM, QDA, and RC (AUC: 0.901–0.906). The combined models outperformed the radiomics model (DeLong test, *p* < 0.001) (Figure 2 and Table S5).

#### 3.2.2 Prediction Model

In discriminating MCI‐s from MCI‐p using radiomics feature‐based models, 10 radiomics features were selected. Among the 10 key features, several first‐order intensity features, including T1_wavelet.LHL_firstorder_Skewness, T1_lbp.3D.m1_firstorder_Maximum, and T1_exponential_firstorder_Maximum, characterize the distribution of voxel intensities within the CP, suggesting the presence of localized high‐intensity regions and asymmetric gray‐level distributions, potentially reflecting variations in tissue composition or microstructural properties. The remaining features are texture‐based, capturing spatial heterogeneity and increased structural complexity of the CP, with some features derived from gradient‐transformed images reflecting variations in local intensity transitions. Overall, the CP of MCI‐p patients appears to exhibit skewed gray‐level distributions, heterogeneous texture, and local signal enhancement, which may serve as quantitative imaging indicators for identifying individuals at higher risk of progression from MCI to AD. The SHAP analysis was presented in Figure S2.

In distinguishing MCI‐p from MCI‐s, model performance was variable, and plsRglm attained the highest AUC (0.745; 95% CI: 0.547–0.911) with precision, recall, and specificity of 0.687, 0.920, and 0.571, respectively. LASSO showed moderate AUC (0.740; 95% CI: 0.550–0.908) with precision, recall, and specificity of 0.684, 0.929, and 0.571, respectively. Overall, classifiers exhibited limited generalizability (AUCs: 0.653–0.745), highlighting the challenge of progression prediction using imaging radiomics features alone (Figure 2 and Table S3).

The integration of clinical and radiomics features appeared to enhance accuracy for predicting the progression of MCI to AD. Within our cohort, the LDA model demonstrated the highest performance, achieving an AUC of 0.900 (95% CI: 0.744–1.000), with a precision of 0.818, recall of 0.900, and specificity of 0.833. It is noteworthy that several other models, including SVM, QDA, and plsRglm, also attained high AUCs of 0.900. Furthermore, models like Enet and LASSO showed consistent and well‐balanced metrics across precision (0.900), recall (0.900), and specificity (0.917) (Figure 2 and Table S5). The optimal performance metrics of the clinical models, CP radiomics models, and combined models for distinguishing MCI from AD and predicting MCI conversion were shown in Table 2.

**TABLE 2:** Performance metrics of the optimal classification and prediction model.

Models	AUC (95% CI)	Precision	Recall	Specificity	Classifier
**AD vs. MCI**					
Clinical	0.809 (0.739–0.872)	0.727	0.810	0.739	plsRglm
CP radiomics	0.794 (0.741–0.847)	0.727	0.688	0.768	SVM
Combined	0.907 (0.848–0.953)	0.885	0.754	0.885	SVM
**MCI‐s vs. MCI‐p**					
Clinical	0.776 (0.592–0.931)	0.684	0.929	0.571	RF
CP radiomics	0.745 (0.547–0.911)	0.687	0.920	0.571	plsRglm
Combined	0.900 (0.744–1.000)	0.818	0.900	0.833	LDA

Abbreviations: AD, Alzheimer's disease; AUC, area under the curve; CI, confidence interval; LDA, Linear Discriminant Analysis; MCI, mild cognitive impairment; MCI‐p, MCI‐progression; MCI‐s, MCI‐stable; plsRglm, Partial Least Squares Regression Generalized Linear Model; RF, Random Forest; SVM, Support Vector Machine.

We further integrated the CP Radscore into the clinical model to construct a combined model, with the corresponding nomogram illustrated in Figure S4. The IDI value was 0.065 (95% CI: 0.038–0.092, *p* < 0.001), and the NRI value was 0.280 (95% CI: 0.102–0.458, *p* = 0.002), both of which confirmed that incorporating the CP Radscore significantly improved the model's accuracy in predicting the progression of MCI to AD, compared to the clinical model alone. Consequently, the nomogram demonstrated significantly superior predictive performance over the clinical model. For details on the classification and prediction models utilizing clinical information alone, please refer to Table S6.

### 3.3 Survival Analysis

**FIGURE 3:**
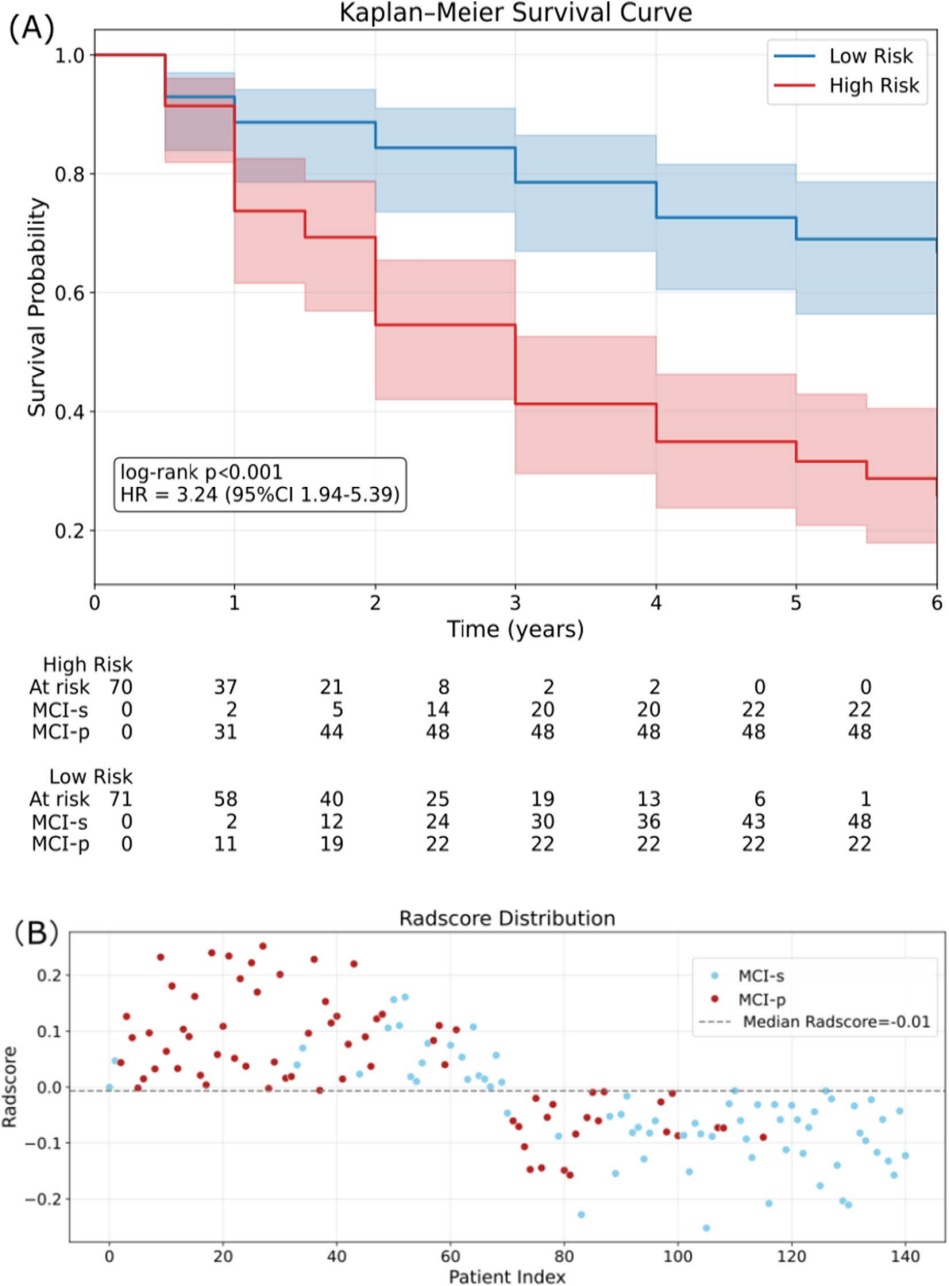
Prognostic value of the Radscore for MCI progression. (A) Kaplan–Meier curves. Patients were dichotomized into high‐risk (red) and low‐risk (blue) groups using the median Radscore as the cut‐off. Shaded areas represent 95% confidence intervals. The number of patients at risk is shown below the plot. A log‐rank test was used to compare survival distributions. The adjusted HR and its 95% confidence interval were derived from univariable Cox regression. (B) Distribution of the Radscore across the entire cohort. Patients are sorted first by Radscore group and then by follow‐up time within each group. Each dot represents one patient; red indicates MCI‐p, blue indicates MCI‐s. HR, hazard ratio; MCI, mild cognitive impairment; MCI‐p, MCI‐progression; MCI‐s, MCI‐stable.

To evaluate the predictive performance for MCI‐to‐AD conversion, patients were stratified into high‐ and low‐risk groups using the median Radscore (−0.01). Kaplan–Meier survival curves were constructed with a median follow‐up of 4 years. The log‐rank test revealed a significant distinction between the two groups (*p* < 0.001), with the high‐risk cohort exhibiting a markedly shorter median progression‐free survival (Figure 3A). To quantify this risk, a Cox proportional‐hazards regression model, adjusted for age, sex, and APOE ε4 status, was employed. It demonstrated that the high‐risk subgroup had a significantly increased hazard of progression, with an adjusted HR of 3.24 (95% CI: 1.94–5.39). Finally, to visualize the stratification, Radscore of each patient was plotted alongside their survival status and time (Figure 3B).

### 3.4 Validation of Radiomics Features in the CP Model

**FIGURE 4:**
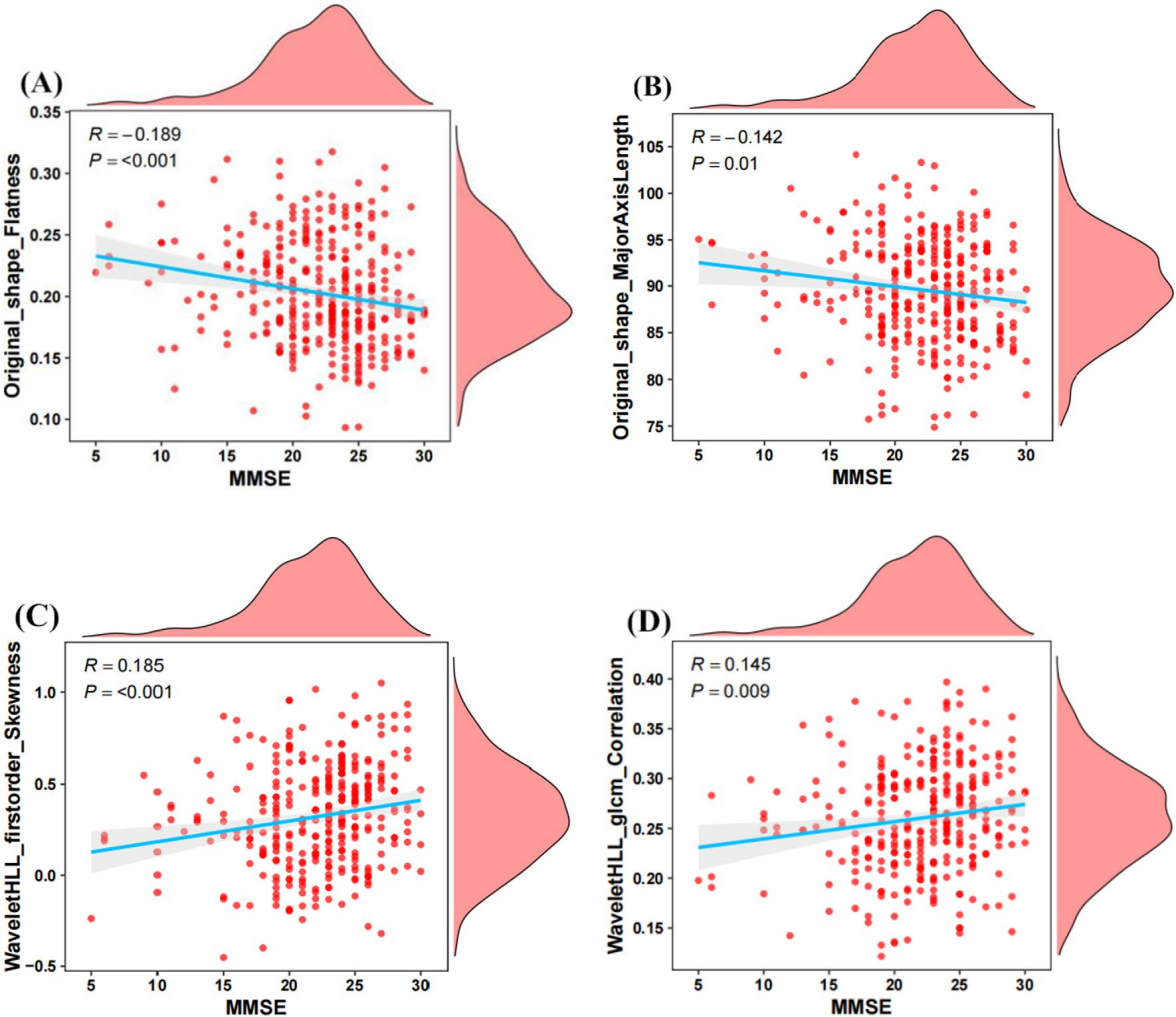
Correlation analysis of CP radiomics features with MMSE scores in subjects with MCI and AD. (A) Original_shape_Flatness, (B) Original_shape_MajorAxisLength, (C) WaveletHLL_firstorder_Skewness, (D) WaveletHLL_glcm_Correlation were significantly correlated with MMSE scores in subjects with MCI and AD. AD, Alzheimer's disease; CP, choroid plexus; MCI, mild cognitive impairment; MMSE, Mini‐Mental State Examination.

The CP radiomics features were significantly correlated with MMSE scores in all patients (adjusted for age, sex, and APOE ε4 status, *p* < 0.05). Key CP radiomics like Original_shape_Flatness (*r* = −0.189, *p* < 0.001), Original_shape_MajorAxisLength (*r* = −0.142, *p* = 0.010), WaveletHLL_firstorder_Skewness (*r* = 0.185, *p* < 0.001), and WaveletHLL_glcm_Correlation (*r* = 0.145, *p* = 0.009) are correlated with MMSE scores (Figure 4).

**FIGURE 5:**
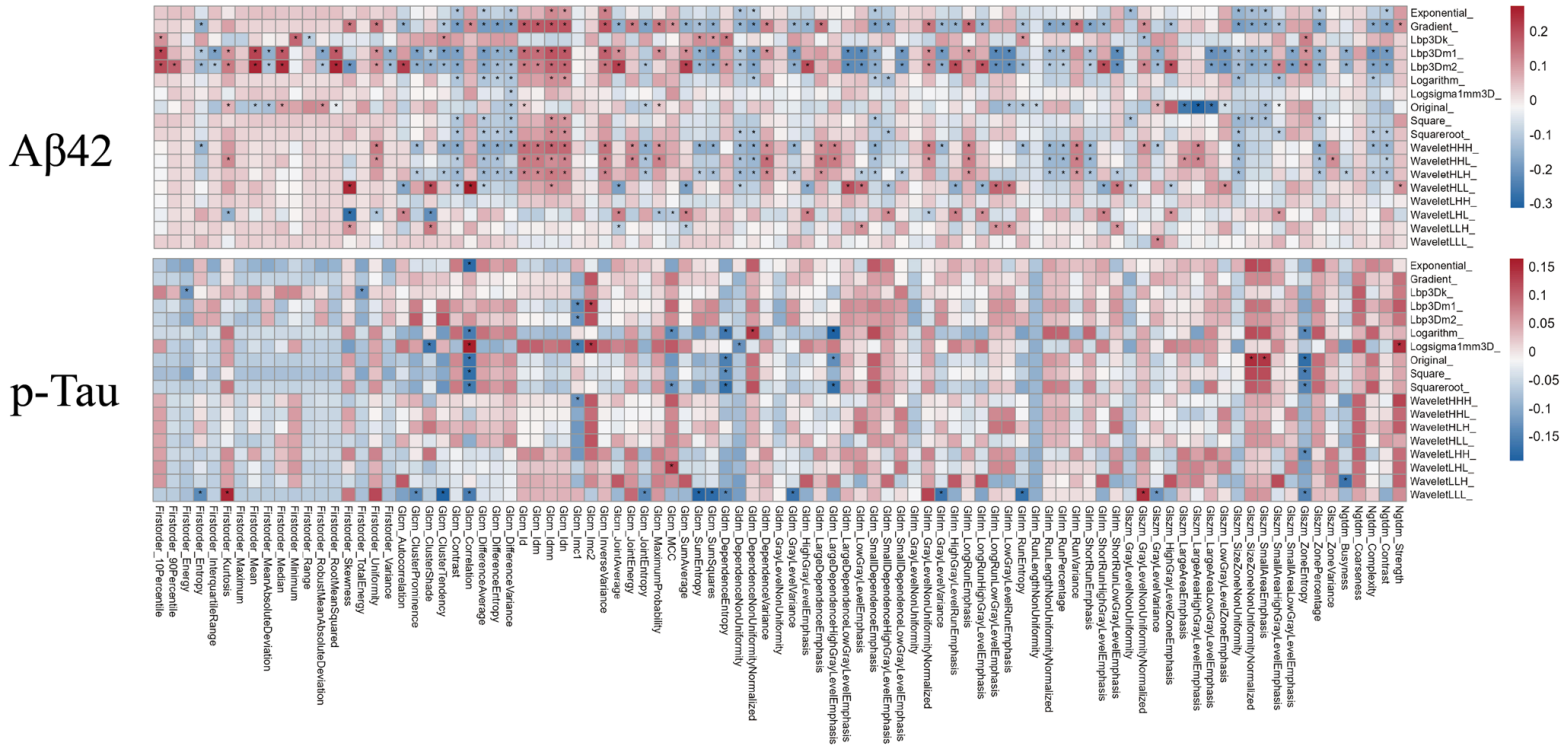
Correlations between CP radiomics features and CSF biomarkers. The heatmap depicts Pearson correlation coefficients (*r*), with red and blue indicating positive and negative associations, respectively. Deeper color shades represent stronger correlations. An asterisk (*) denotes correlations that remained significant after FDR correction at *p* < 0.05. Aβ42, amyloid‐beta 42; CP, choroid plexus; CSF, cerebrospinal fluid; FDR, false discovery rate; p‐Tau, phosphorylated tau.

Correlation analyses were conducted to examine the associations between CP radiomics features and AD pathological biomarkers, specifically CSF levels of Aβ42 and p‐Tau. Among the CP radiomics features, 358 exhibited significant correlations with Aβ42 levels (*p* < 0.05), while 51 features showed significant correlations with CSF p‐Tau levels (*p* < 0.05) as shown in Figure 5.

**FIGURE 6:**
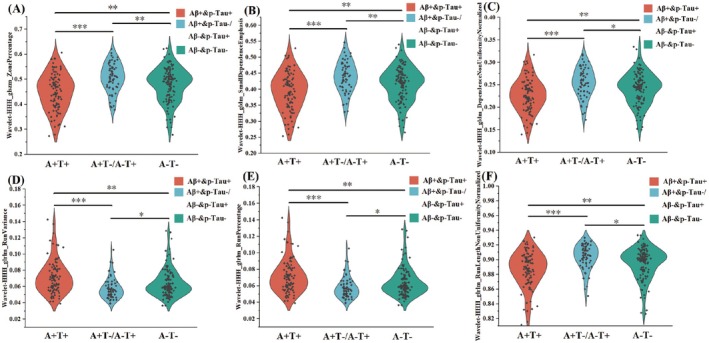
Comparison of representative CP radiomics features across different Aβ and p‐Tau pathology stages in AD and MCI. The following features were assessed across the Aβ/p‐Tau subgroups (A+T+, A+T−/A−T+, and A−T−): (A) Wavelet‐HHH_glszm_ZonePercentage, (B) Wavelet‐HHH_gldm_SmallDependenceEmphasis, (C) Wavelet‐HHH_gldm_DependenceNonUniformityNormalized, (D) Wavelet‐HHH_glrlm_RunVariance, (E) Wavelet‐HHH_glrlm_RunPercentage, and (F) Wavelet‐HHH_glrlm_RunLengthNonUniformityNormalized. *p < 0.05; **p < 0.01; ***p < 0.001. A+T−/A−T+, Aβ+&p‐Tau−/Aβ−&p‐Tau+; A+T+, Aβ+&p‐Tau+; AD, Alzheimer's disease; A−T−, Aβ−&p‐Tau−; Aβ, amyloid beta; CP, choroid plexus; MCI, mild cognitive impairment; p‐Tau, phosphorylated tau.

We further analyzed CP radiomics features across various stages of Aβ42 and p‐Tau status, defining the Aβ+ group as Aβ42 < 651 (mean − 0.5 × standard deviation [SD]) and the Aβ− group as Aβ > 1184 (mean + 0.5 × SD). Based on CSF p‐Tau levels, participants were stratified as p‐Tau+ (> 31 pg/mL; mean + 0.5 × SD) or p‐Tau− (< 22 pg/mL; mean − 0.5 × SD). Comparisons across the resulting biomarker subgroups (Aβ−&p‐Tau−, Aβ+&p‐Tau−, Aβ−&p‐Tau+, and Aβ+&p‐Tau+) demonstrated significant differences in CP radiomics features (Table S7). Notable changes were observed in Wavelet‐HHH_glszm_ZonePercentage and Wavelet‐HHH_gldm_SmallDependenceEmphasis, Wavelet‐HHH_gldm_DependenceNonUniformityNormalized, Wavelet‐HHH_glrlm_RunVariance, Wavelet‐HHH_glrlm_RunPercentage and Wavelet‐HHH_glrlm_RunLengthNonUniformityNormalized (Figure 6).

## 4 Discussion

This study developed and validated machine learning models based on CP radiomics features for distinguishing AD from MCI and predicting MCI progression to AD. Importantly, the integration of clinical information markedly improved model performance, yielding AUCs exceeding 0.900 for both diagnostic and prognostic tasks. These results highlight the synergistic benefit of combining imaging biomarkers with clinical data and support CP radiomics as a non‐invasive tool for the identification of prodromal AD detection and risk stratification. Moreover, the significant associations between key CP radiomics features, CSF Aβ/p‐Tau levels, and MMSE scores suggest that CP radiomics may reflect underlying AD‐related pathophysiological changes. Overall, this study underscores the value of CP radiomics as a complementary diagnostic biomarker and provides insights into the mechanisms underlying CP pathological alterations in AD.

The CP is a highly vascularized structure located in the ventricles of the brain and is responsible for the production of CSF [9]. It is composed of a monolayer of epithelial cells that form the blood‐CSF barrier, regulating the exchange of molecules between the blood and the CSF [25, 26]. In AD, the CP undergoes various morphological and functional changes, including epithelial atrophy, thickening of the basement membrane, and stroma fibrosis [25]. These alterations can lead to decreased CSF turnover and impaired barrier function, contributing to the disruption of brain homeostasis and the progression of AD pathology [26]. Importantly, the CP plays a crucial role in the clearance and transport of Aβ peptides, which are a hallmark of AD pathology [27]. Impairment of the CP's ability to clear Aβ from the CSF and transport it across the blood‐CSF barrier has been linked to the accumulation of Aβ in the brain, a key event in the development of AD [28]. This study revealed that CP texture features, such as Wavelet‐HHH_glszm_ZonePercentage and Wavelet‐HHH_gldm_SmallDependenceEmphasis, exhibited significant differences across various Aβ and p‐Tau stages, indicating that microstructural changes in the CP are closely associated with AD pathological markers. These findings align with previous studies demonstrating that the CP participates in Aβ clearance through the tight junctions and transport functions of its epithelial cells [29]. Further analysis demonstrated that CP morphological features were significantly correlated with Aβ42 levels, supporting the role of CP in Aβ protein clearance [30]. Structural changes in the CP, such as epithelial atrophy and thickening of the basement membrane, may worsen abnormal Aβ deposition by impairing the function of the blood‐CSF barrier [28]. These findings provide imaging evidence for the CP as a target for pathological monitoring across the AD continuum and suggest that CP radiomics features could serve as non‐invasive biomarkers.

Furthermore, this study found that CP radiomics features were significantly correlated with cognitive function and clinical prognosis across the AD continuum. For instance, Wavelet‐HLL_firstorder_Skewness exhibited a positive correlation with MMSE scores. The results suggest that CP microstructural heterogeneity may directly contribute to the pathophysiological process of cognitive decline by influencing CSF dynamics and metabolic waste clearance efficiency [27]. In predicting MCI‐to‐AD conversion, high‐risk patients demonstrated CP features characterized by reduced texture complexity and increased morphological irregularity, which were significantly associated with shorter AD conversion times. Consistent with previous studies, CP dysfunction may lead to the accumulation of neuroinflammatory factors and toxic proteins, subsequently triggering synaptic damage and neuronal death [25, 26].

Although the clinical model slightly outperformed the CP radiomics model in distinguishing AD from MCI, this difference likely reflects the direct association of established clinical biomarkers, such as CSF Aβ42, p‐Tau, and APOE ε4 status, with core AD pathology and their long‐validated diagnostic stability [31]. In contrast, CP radiomics features non‐invasively capture microstructural alterations of CP [32], but their relationship with AD pathology requires indirect inference and may be influenced by MRI acquisition parameters and segmentation accuracy, partly limiting standalone performance. Nevertheless, the clinical value of CP radiomics extends beyond absolute diagnostic accuracy. Its major advantage lies in timely non‐invasive screening, particularly for patients who face difficulties with CSF collection, and in detecting subtle texture abnormalities that may precede conventional volumetric atrophy [33]. Importantly, the significant associations between CP radiomic features and Aβ42/p‐Tau provide imaging evidence supporting the hypothesis that blood‐CSF barrier dysfunction plays a key role in AD progression [9], highlighting the CP as a potential mechanistic biomarker and intervention target.

Compared with hippocampal‐based models, which typically achieve AUC values of 0.80–0.85 using volumetric measurements and up to 0.85–0.90 when advanced radiomics or deep learning methods are applied [34, 35], the CP model demonstrates slightly inferior diagnostic performance. This discrepancy, even for the integrated model combining CP radiomics with clinical markers, likely reflects the pathological cascade of AD. While hippocampal features represent a definitive downstream structural endpoint, CP features capture upstream pathological processes, such as impaired protein clearance and barrier dysfunction [36]. These early‐stage alterations manifest as more subtle and heterogeneous microstructural changes, resulting in smaller effect sizes compared to overt structural atrophy. Consequently, the discriminative power in classification tasks is driven more by the pathological directness of the markers, where overt neurodegeneration remains the most robust predictor, rather than the sheer diversity or number of features included in the integrated model [37]. Furthermore, while hippocampal atrophy is widely recognized as a key marker of neurodegeneration within the AT(N) framework (amyloid, tau, and neurodegeneration) and is commonly observed in patients with established AD [38], CP alterations are not directly tied to diagnostic classification but instead reflect broader pathophysiological processes, including inflammation and barrier dysfunction, that evolve gradually across the AD continuum [11]. As a result, hippocampal features are more proximally linked to diagnostic labels, which may contribute to their superior performance in classification tasks. Importantly, this does not diminish the clinical relevance of CP imaging biomarkers. On the contrary, their ability to reflect early and upstream pathological changes suggests a complementary role to hippocampal markers, particularly in the prodromal stages of AD, where overt neurodegeneration may not yet be detectable. Moreover, the observed correlations between CP features, cognitive scores, and MCI‐to‐AD conversion indicate that CP radiomics may be suitable for disease monitoring and risk stratification.

This study has several limitations. First and foremost, while our AD/MCI classification was externally validated in an independent Chinese cohort, the longitudinal progression model lacks an independent external validation. This is primarily due to the scarcity of clinical cohorts that possess long‐term follow‐up data coupled with confirmed amyloid and tau biomarker status according to the National Institute on Aging and the Alzheimer's Association (NIA‐AA) framework. Secondly, the reliance on retrospective data from specific cohorts may limit generalizability to broader populations. Thirdly, CP‐based radiomics has not yet been incorporated into routine clinical workflows for the diagnosis or prediction of MCI and AD, which may limit its immediate clinical applicability. The calculation of Radscore still depends on image processing and computational pipelines, and its integration into routine clinical practice may require further technical development and standardization. Fourthly, the CP segmentation and radiomics feature extraction could be influenced by MRI protocol variability. Moreover, while the CP model performance improved with clinical features, its standalone diagnostic accuracy remains moderate. Future studies should prospectively validate the model in diverse, real‐world cohorts, standardize imaging protocols, and explore multimodal integration to enhance predictive power. In addition, the small sample size of longitudinal data may compromise the model's reliability. Lastly, although the results are promising and suggest a potential synergistic value of combining clinical and radiomic data for predicting the progression of MCI to AD, the wide confidence intervals warrant caution in interpreting their generalizability. Future research should incorporate larger longitudinal cohorts to verify the model's accuracy in tracking disease progression and aiding clinical decision‐making.

This study constructs a machine learning model based on the imaging features of CP, which effectively distinguishes AD patients from those with MCI and predicts the risk of MCI conversion to AD. When combined with clinical features, the model's performance significantly improves, providing a non‐invasive tool for prodromal AD identification and risk stratification. The study reveals the correlation between CP radiomics features and disease severity as well as pathological biomarkers, offering a new perspective on exploring the role of CP in the pathological mechanisms of AD.

## Author Contributions

**Feiyue Yin:** writing – review and editing, writing – original draft, visualization, validation, software, resources, methodology, investigation, funding acquisition, formal analysis, data curation, conceptualization. **Xiaohua Wang:** formal analysis, data curation, validation. **Xiao Chen:** data curation, methodology. **Jinju Sun:** data curation, methodology. **Kai Zhang:** methodology. **Xiaojuan Dong:** data curation, software. **Wenwen Wang:** data curation. **Peng Zeng:** resources. **Binglan Li:** resources. **Lisha Nie:** resources. **Dan Luo:** resources. **Yongmei Li:** writing – review and editing, supervision, resources, project administration, funding acquisition, formal analysis, conceptualization. **Tianyou Luo:** writing – review and editing, supervision, resources, project administration, funding acquisition, formal analysis, conceptualization.

## Funding

This work was supported by National Institute on Aging (National Institutes of Health Grant U19AG024904), Chongqing Science and Technology Bureau (cstc2022ycjh‐bgzXM0230 to T.L.), Chongqing Medical Scientific Research project (Joint project of Chongqing Health Commission and Science and Technology Bureau) (2023ZDXM006 to Y.L.), the Key Project of Technological Innovation and Application Development of Chongqing Science and Technology Bureau (CSTC2021 jscx‐gksb‐N0008 to T.L.) and Doctoral Innovation Research Project of the First Clinical College, Chongqing Medical University (CYYY‐BSCX202506 to F.Y.).

## Ethics Statement

The study protocol was approved by the ethics committee of Daping Hospital of Army Medical University (No. 2025‐350).

## Consent

In‐house participants provided written informed consent for publication of the data contained in this study.

## Conflicts of Interest

The authors declare no conflicts of interest.

## Supporting information


**Figure S1:** Example choroid plexus segmentations using UX‐Net in patients with MCI and AD from ADNI dataset and in‐house dataset.
**Figure S2:** Interpretation of radiomics features from MCI/AD classification to progression prediction using SHAP analysis.
**Figure S3:** ROC curves of the classification models for discriminating AD from MCI in the external validation cohort.
**Figure S4:** Nomogram prediction and validation for MCI‐to‐AD conversion.
**Table S1:** Segmentation accuracy of 3D UX‐Net compared to manual annotations.
**Table S2:** Segmentation accuracy of 3D UX‐Net Compared To Manually Refined Masks.
**Table S3:** Performance metrics of radiomics feature‐based classification models.
**Table S4:** Performance metrics of classification models in the external validation cohort.
**Table S5:** Performance metrics of clinical and radiomics feature‐based classification models.
**Table S6:** Performance metrics of clinical feature‐based classification models.
**Table S7:** Comparison of representative CP radiomic features across different Aβ and p‐Tau pathology stages.
**Method S1:** Cohort selection, diagnostic criteria, and biomarker assessment.
**Method S2:** Automatic choroid plexus segmentation using 3D UX‐Net.
**Method S3:** Radiomics feature extraction and normalization.
**Method S4:** Multi‐stage feature selection pipeline.
**Method S5:** Machine learning classification and model evaluation.
**Method S6:** Model interpretation via SHAP analysis.
**Method S7:** Nomogram construction.

## Data Availability

The datasets analyzed during the current study are available in Alzheimer's Disease Neuroimaging Initiative (http://adni.loni.usc.edu). Anonymized data used in this study from Daping Hospital of Army Medical University are available upon reasonable request from the corresponding authors.
