# Supplementary material for: Decoding Choroid Plexus Pathology in Alzheimer's Disease: A Longitudinal Radiomics Approach for Prodromal Identification and Risk Stratification
Source: CNS Neurosci Ther. 2026 Jun 15;32(6):e70987. doi: 10.1002/cns.70987 (PMC13269847; doi:10.1002/cns.70987)
Supplement: Supplementary file 1 — Figure S1: Example choroid plexus segmentations using UX‐Net in patients with MCI and AD from ADNI dataset and in‐house dataset. Figure S2: Interpretation of radiomics features from MCI/AD classification to progression prediction using SHAP analysis. Figure S3: ROC curves of the classification models for discriminating AD from MCI in the external validation cohort. Figure S4: Nomogram prediction and validation for MCI‐to‐AD conversion. Table S1: Segmentation accuracy of 3D UX‐Net compared to manual annotations. Table S2: Segmentation accuracy of 3D UX‐Net Compared To Manually Refined Masks. Table S3: Performance metrics of radiomics feature‐based classification models. Table S4: Performance metrics of classification models in the external validation cohort. Table S5: Performance metrics of clinical and radiomics feature‐based classification models. Table S6: Performance metrics of clinical feature‐based classification models. Table S7: Comparison of representative CP radiomic features across different Aβ and p‐Tau pathology stages. Method S1: Cohort selection, diagnostic criteria, and biomarker assessment. Method S2: Automatic choroid plexus segmentation using 3D UX‐Net. Method S3: Radiomics feature extraction and normalization. Method S4: Multi‐stage feature selection pipeline. Method S5: Machine learning classification and model evaluation. Method S6: Model interpretation via SHAP analysis. Method S7: Nomogram construction. [file CNS-32-e70987-s001.docx]

**Decoding Choroid Plexus Pathology in Alzheimer's Disease: A Longitudinal Radiomics Approach for Prodromal Identification and Risk Stratification**

**Supplementary Method**

Method S1

A primary cohort of 898 subjects from the Alzheimer's Disease Neuroimaging Initiative (ADNI) database was utilized, comprising 472 with MCI and 426 with AD. This study included participants enrolled across the ADNI-1, ADNI-GO, ADNI-2, and ADNI-3 cohorts (http://adni.loni.usc.edu/, accessed 19 October 2025) and underwent Apolipoprotein E (APOE) genotyping and comprehensive clinical assessments, including the Mini-Mental State Examination (MMSE) and the Clinical Dementia Rating (CDR). Among them, a subset of 622 subjects had quantifiable CSF biomarker data (Aβ42, total tau, and p-tau) measured at the University of Pennsylvania Biomarker Research Laboratory. Participants from the ADNI cohorts (ADNI-1, GO, 2, and 3) were categorized into AD and MCI groups based on standardized clinical criteria. Patients with AD were required to have MMSE scores between 20–26, a CDR of 0.5 or 1.0, and meet the National Institute of Neurological and Communicative Disorders and Stroke and the Alzheimer’s Disease and Related Disorders Association (NINCDS/ADRDA) criteria for probable AD. Subjects with MCI were characterized by MMSE scores of 24–30, a subjective memory complaint, a CDR of 0.5 (with a memory box score ≥0.5), and objective memory loss as measured by education-adjusted scores on the Wechsler Memory Scale Logical Memory II (WMS-LM II). These diagnostic criteria were largely consistent across ADNI phases, with only minor refinements, ensuring the compatibility of the combined dataset for robust model training and internal validation. The diagnostic criteria for AD and MCI in the ADNI cohort are detailed at https://adni.loni.usc.edu/help-faqs/adni-documentation/. Accessed 19 October 2025.

A total of 232 out of the 472 MCI participants completed a 72-month follow-up period. In the initial three years, evaluations were conducted every 6–12 months, followed by annual assessments thereafter. During follow-up, 100 MCI demonstrated progression from MCI to AD as defined by the National Institute on Aging and the Alzheimer's Association (NIA-AA) framework, requiring: (1) a clear progression in clinical stage, evidenced by a change in CDR global score from 0.5 at baseline to 1.0 or greater, consistent with a clinical diagnosis of dementia, and (2) biomarker confirmation of Alzheimer's pathology through either CSF analysis or PET. Based on longitudinal outcomes, MCI were categorized into: (1) MCI-stable (MCI-s) (n=132), who retained their baseline diagnosis, and (2) MCI-progressed (MCI-p) (n=100), who converted to AD. Figure 1 shows the study flowchart. Due to a significant baseline age difference between the MCI-s and MCI-p groups, propensity score matching (PSM) based on age and sex was performed. This resulted in a matched cohort of 71 MCI-s and 71 MCI-p patients for progression analysis.

An independent cohort of 150 subjects including 70 MCI and 80 AD from Daping Hospital of Army Medical University was included for external validation. All individuals in the cohort had plasma samples for assays and underwent Aβ and/or tau PET scans within 1 year of blood collection between January 2015 and May 2024. The inclusion criteria were as follows : (1) Patients with AD were included if they had a CDR score of 1 to 3, and had positive Aβ PET and Tau PET scans according to 2023 Alzheimer’s Association diagnostic criteria. (2) Individuals with MCI were required to exhibit both subjective and objective cognitive impairment, with a CDR score of 0.5, while not meeting the diagnostic criteria for dementia (based on DSM-5). This clinical definition is fundamentally consistent with the ADNI recruitment criteria, ensuring that the diagnostic categories are conceptually aligned and clinically comparable, although not identical. The exclusion criteria were as follows: (1) A diagnosis of MCI attributable to other neurological disorders, such as Parkinson's disease, vascular dementia, or Lewy body disease; (2) the presence of other major psychiatric or systemic illnesses that could significantly confound cognitive assessment. In the external validation cohort, clinical assessments (MMSE and CDR) followed ADNI criteria; however, the corresponding PET scans and plasma samples, though conducted, are not available for disclosure due to data protection agreements.

The study protocol was approved by the ethics committee of Daping Hospital of Army Medical University (No.2025-350) and conducted according to the Declaration of Helsinki. All study participants provided written informed consent prior to their participation. The imaging parameters were: T1 Magnetization Prepared Rapid Acquisition Gradient Echo (T1-MPRAGE): repetition time = 7900 ms, echo time = 4.0 ms, flip angle = 9°, thickness = 1mm, field of view = 256 × 256, matrix = 256 × 256.

Method S2

For the automatic segmentation of the CP, we employed the pre-trained 3D UX-Net framework, as originally proposed and validated by Wang et al^[1]^. This architecture is specifically designed for volumetric medical image segmentation and captures long-range spatial dependencies using large-kernel convolutions, mimicking certain characteristics of transformer-based models^[1,2]^.

The core of the segmentation pipeline relies on the 3D UX-Net block, which enables a substantial effective receptive field through depth-wise convolution (DWC) with a large kernel size of 7×7×7. Additionally, depthwise convolutional scaling (DCS) with a 1×1×1 kernel is employed to linearly scale channel-wise features, contributing to improved feature representation while reducing redundant information across channels.

The architecture adopts a U-shaped encoding–decoding structure with long skip connections to preserve high-resolution spatial information. Layer normalization and Gaussian Error Linear Unit (GELU) activation are utilized to improve computational efficiency and numerical stability.

In this study, we directly utilized the pre-trained model weights provided by Wang et al. without additional training or fine-tuning^[1]^. The model was originally optimized using the Dice loss function to address class imbalance, as the CP occupies only a small fraction of the intracranial volume. The Dice loss is mathematically defined as:

$$D=1-\frac{2\sum_{i=1}^{N} p_{i}g_{i}+\epsilon}{\sum_{i=1}^{N} p_{i}+\sum_{i=1}^{N} g_{i}+\epsilon}$$

where $p_{i}$ and $g_{i}$ represent the predicted and ground truth values, respectively, and $\epsilon$ is a smoothing term for numerical stability.

All input images were standardized using Z-score normalization to ensure consistency with the original training configuration. A sliding window inference strategy was applied to process the volumetric data efficiently while maintaining segmentation performance^[3]^. By leveraging this pre-trained volumetric network, we achieved automated and reproducible quantification of CP volumes across the dataset.

1. Wang X, Wang X, Yan Z, et al. Enhanced choroid plexus segmentation with 3D UX-Net and its association with disease progression in multiple sclerosis. Mult Scler Relat Disord. 2024 Aug;88:105750. doi: 10.1016/j.msard.2024.105750.
2. Lee H.H., Bao S., Huo Y., et al. 3D UXNet: a large kernel volumetric convnet modernizing hierarchical transformer for medical image segmentation[Z]. the Kigali Convention Center:OpenReview.net,2023.
3. Yazdan-Panah A, Schmidt-Mengin M, Ricigliano VAG, et al. Automatic segmentation of the choroid plexuses: Method and validation in controls and patients with multiple sclerosis. Neuroimage Clin. 2023;38:103368. doi: 10.1016/j.nicl.2023.103368.

Method S3

Radiomics features were extracted from the original T1-MPRAGE images based on the finalized CP masks. We extracted three categories of features from the original images: first-order statistics, shape, and texture features. First-order statistics describe the distribution of voxel intensities within the CP region, including metrics such as mean, standard deviation, skewness, and kurtosis. Shape features were extracted to characterize the geometric attributes of the CP, encompassing volume, surface area, and compactness. Texture features, which reflect spatial patterns and voxel intensity relationships, were calculated based on the following matrices: gray-level co-occurrence matrix (GLCM), gray-level run-length matrix (GLRLM), gray-level size zone matrix (GLSZM), gray-level dependence matrix (GLDM), and neighborhood gray-tone difference matrix (NGTDM). These matrices yield specific texture metrics such as contrast, correlation, and homogeneity. In addition to the original image, we also applied wavelet and Laplacian of Gaussian (LoG) filter transformations to generate derived images. Texture and first-order features were subsequently extracted from these transformed images as well. In total, this comprehensive process yielded 1,688 radiomics features for each subject. Ultimately, Z-score normalization was used to equalize all features, guaranteeing a mean of 0 and a standard deviation of 1.

Method S4

A multi-stage feature selection pipeline was employed to identify the most predictive features. Initially, we selected features with statistically significant discriminative power (*p* < 0.05) using either the Mann-Whitney U test or the t-test, based on data distribution characteristics. To ensure feature independence and enhance model generalizability, we subsequently eliminated highly correlated features (Pearson's *r* > 0.9). The remaining features were then refined through Mutual Information analysis, retaining only those with non-zero information gain to improve predictive efficiency. For the final selection, we applied LASSO regression with 5-fold cross-validation to determine the optimal regularization parameter. Features with non-zero regression coefficients at this optimal λ were incorporated into the final model. The LASSO-based selection procedure allows automatic selection of an optimal feature subset, which helps mitigate overfitting and improves model generalizability. This rigorous sequential approach ensured a balance between statistical significance, feature independence, informational value, and predictive relevance.

Method S5

Classification models were constructed to distinguish MCI from AD, as well as to separate MCI-s from progressive MCI-p. Twelve machine learning algorithms were employed: Support Vector Machine (SVM), Random Forest (RF), eXtreme Gradient Boosting (XGBoost), Light Gradient Boosting Machine (LightGBM), Ridge Classifier (RC), Naive Bayes (NB), Linear Discriminant Analysis (LDA), Quadratic Discriminant Analysis (QDA), Gradient Boosting Machine (GBM), Elastic Net (Enet), Partial Least Squares Regression Generalized Linear Model (plsRglm), and least absolute shrinkage and selection operator (LASSO). Using stratified sampling, participants were randomly split into training and test sets at an 8:2 ratio. Model training and hyperparameter optimization were conducted via five-fold cross-validation on the training set, and final performance was assessed on the held-out test set. To mitigate class imbalance, the Synthetic Minority Over-sampling Technique (SMOTE) was applied during training to reduce overfitting and enhance model robustness. Model performance was evaluated using the receiver operating characteristic (ROC) curve, with the area under the curve (AUC) and its 95% confidence interval (95% CI) reported as key metrics.

Method S6

Shapley Additive exPlanations (SHAP) was employed based on the Shapley value from cooperative game theory to analyze the contribution mechanisms of radiomic features to model predictive performance. The method quantifies the marginal contribution of each feature in model decision-making, enabling unified evaluation of both continuous and categorical variables. For visualizing feature importance, SHAP analysis results are typically presented in a vertical ranked format. In this layout, features are arranged along the vertical axis in descending order of importance—from top to bottom. The SHAP visualizations provide a comprehensive view of feature contributions to model predictions. In the bar plot, the horizontal axis represents the mean absolute SHAP values, with larger values indicating greater overall importance of the corresponding feature in influencing the model’s output. The bee swarm plot further illustrates the distribution of SHAP values across all samples, where the horizontal position indicates the contribution of each feature to the prediction (positive values push toward AD or MCI-progression, negative toward MCI or MCI-stable), and the color represents the original feature value (red for high, blue for low). Notably, for some features, higher values may push predictions in either direction depending on the sample, reflecting non-linear or interaction effects captured by the model. Finally, the decision plot traces individual predictions from the baseline to the final output, showing how sequentially adding features shifts the model’s prediction for each sample and highlighting the key features that play a decisive role in individual-level classification.

Method S7

A multivariate logistic regression model combining clinical features and Radscore was constructed to predict the risk of MCI progression to AD. To facilitate clinical applications, we generated a nomogram for MCI-to-AD progression using the logistic regression model, illustrating the contribution of each variable and the method for calculating risk probabilities. To validate the model's stability and accuracy, we plotted calibration curves and performed 200 bootstrap resamples to assess the agreement between predicted probabilities and observed probabilities. Furthermore, a clinical model without Radscore was developed as a baseline for comparison. The Net Reclassification Index (NRI) and Integrated Discrimination Improvement (IDI) were computed to evaluate and compare the discriminative performance between the models.

**Supplementary Figure**

| \| 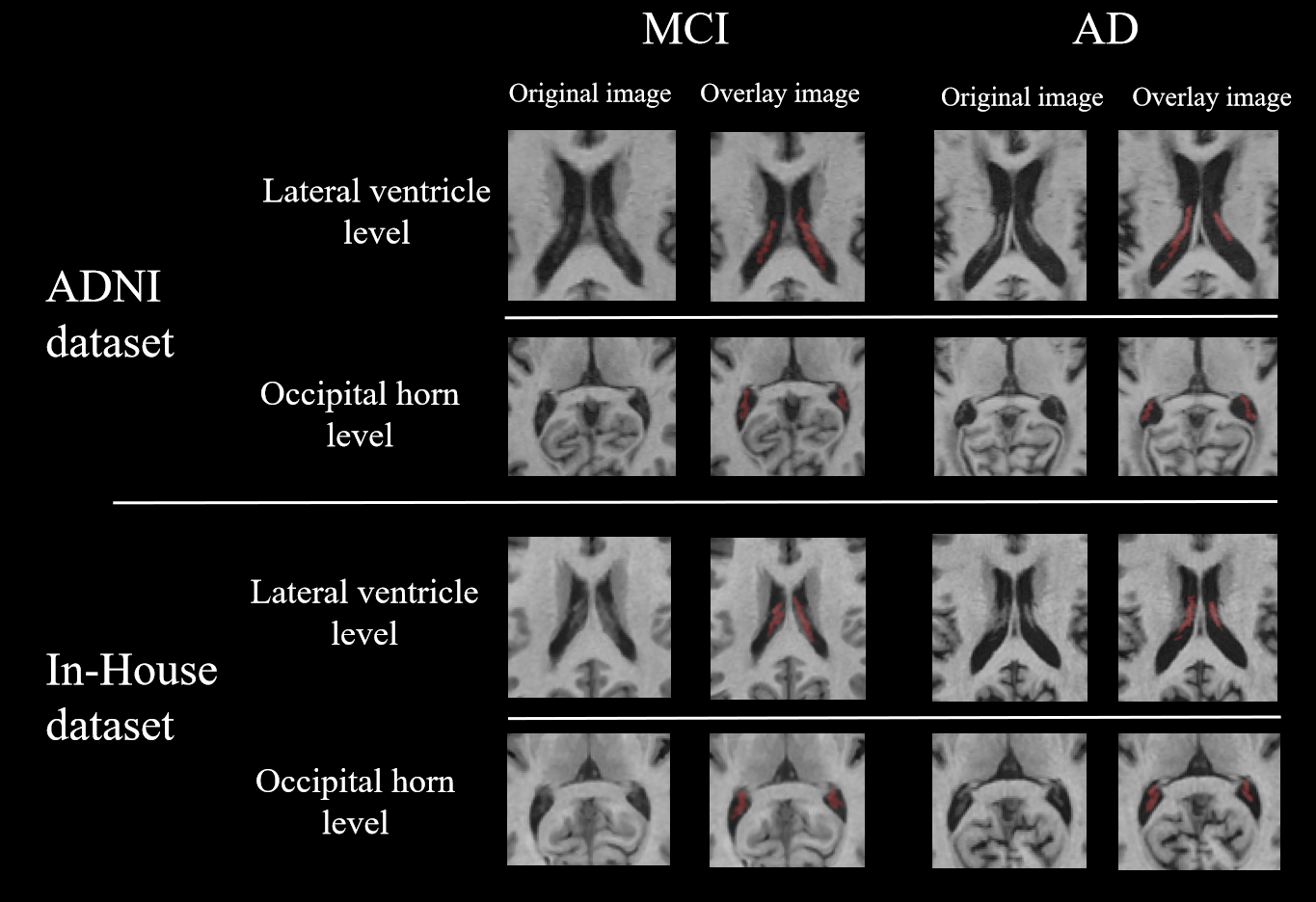 \| \| --- \| \| **Figure S1 Example Choroid Plexus Segmentations using UX-Net in patients with MCI and AD from ADNI dataset and In-house dataset**  Abbreviations: MCI, mild cognitive impairment; AD, Alzheimer's disease; ADNI, Alzheimer's Disease Neuroimaging Initiative. \| |  |
| --- | --- | --- | --- |
| 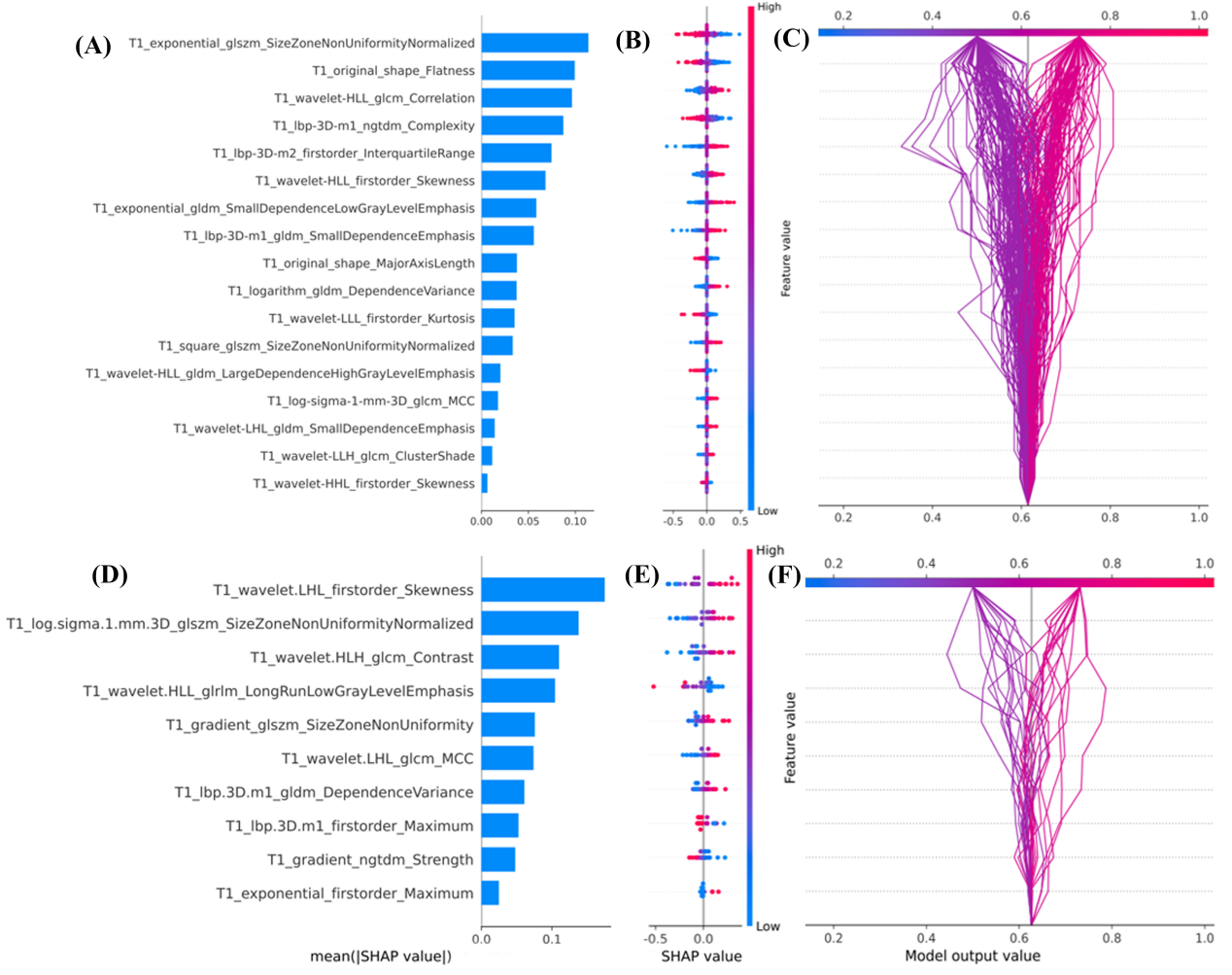 |  |
|  |  |
| **Figure S2 Interpretation of radiomics features from MCI/AD classification to progression prediction using SHAP analysis**  (A-C) SHAP analysis for MCI/AD classification. (A) Feature importance bar plot ranked by mean absolute SHAP values. (B) Bee swarm plot showing SHAP value distribution and directional influence per feature. (C) Decision plot illustrating how features shift individual predictions from baseline.  (D-F) SHAP analysis for MCI-to-AD progression prediction. (D–F) Corresponding plots (bar, bee swarm, and decision plots) quantifying feature contributions to progression risk prediction.  Abbreviations: SHAP, Shapley Additive exPlanations; MCI, mild cognitive impairment; AD, Alzheimer's disease.   \| 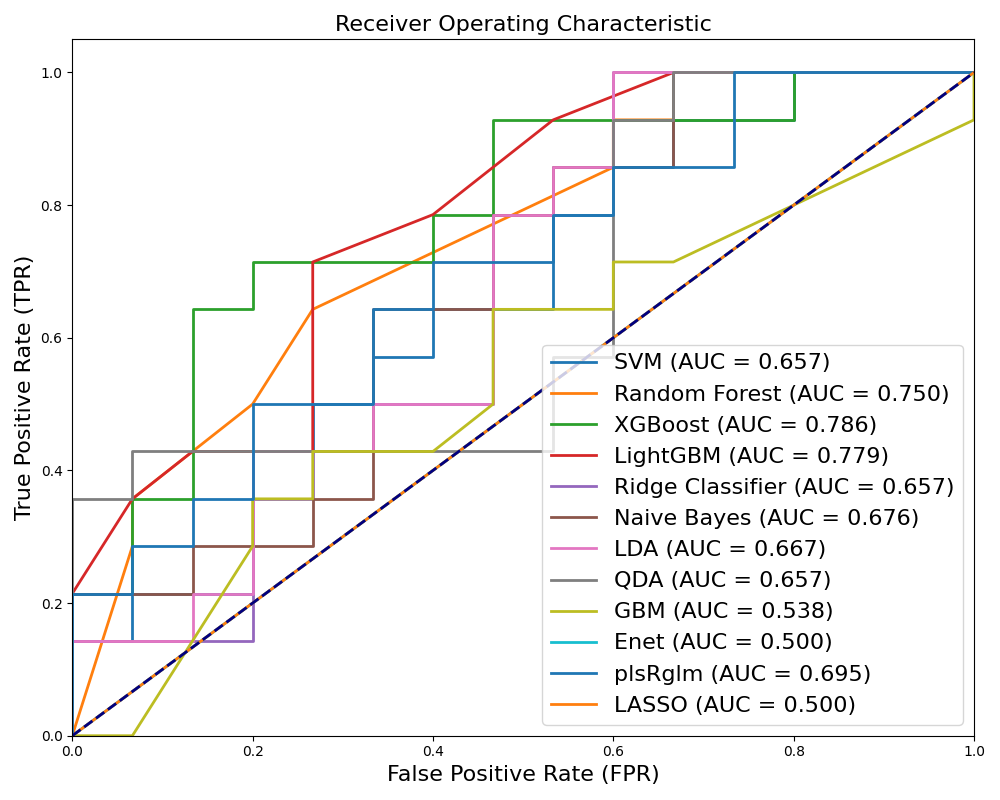 \| \| --- \| \| **Figure S3 ROC curves of the classification models for discriminating AD from MCI in the external validation cohort**  Abbreviations: SVM, Support Vector Machine; RF, Random Forest; XGBoost, eXtreme Gradient Boosting; LightGBM, Light Gradient Boosting Machine; RC, Ridge Classifier; NB, Naive Bayes; LDA, Linear Discriminant Analysis; QDA, Quadratic Discriminant Analysis; GBM, Gradient Boosting Machine; Enet, Elastic Net; plsRglm, Partial Least Squares Regression Generalized Linear Model; LASSO, Least Absolute Shrinkage and Selection Operator, ROC, receiver operating characteristic \| |  |
| \| 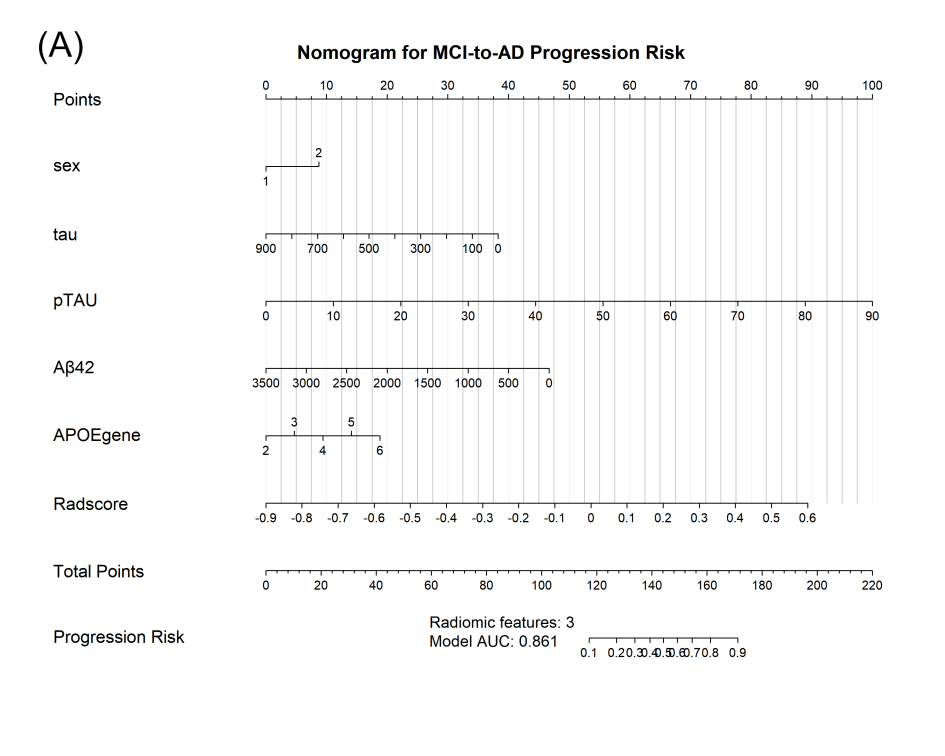 \| \| --- \| \| 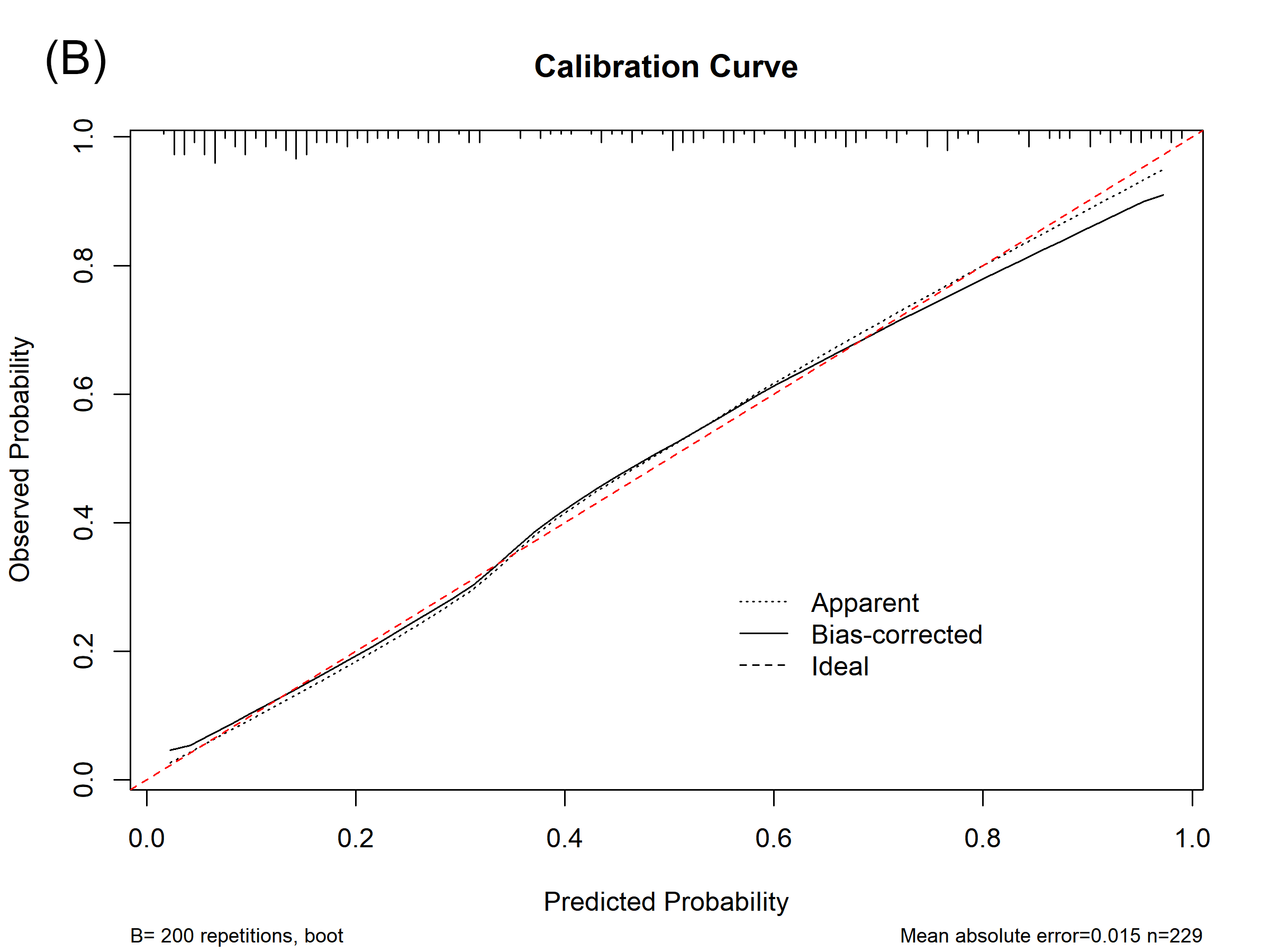 \| \| **Figure S4 Nomogram Prediction and Validation for MCI-to-AD Conversion**   1. Nomogram for predicting the conversion from MCI to AD; (B) Calibration plot of the nomogram for predicting the conversion from MCI to AD.   Abbreviations: MCI, mild cognitive impairment; AD, Alzheimer's disease; p-Tau, phosphorylated tau; Aβ, amyloid-beta; APOE, Apolipoprotein E; AUC, area under the curve. \|  \| **Supplementary Table**   \| **Table S1 Segmentation Accuracy of 3D UX-Net Compared to Manual Annotations** \| \| \| \| \| \| --- \| --- \| --- \| --- \| --- \| \| Group \| Subgroup \| Dice Score \| Jaccard Index \| HD95 \| \| ADNI dataset \| MCI (n=20) \| 0.8299 (0.0881) \| 0.7181 (0.1193) \| 1.58 (0.84) \| \| AD (n=20) \| 0.8422 (0.0914) \| 0.7381 (0.1357) \| 2.01 (1.41) \| \| In-House dataset \| MCI (n=5) \| 0.8728 (0.0359) \| 0.7761 (0.0555) \| 1.08 (0.17) \| \| AD (n=5) \| 0.8104 (0.0522) \| 0.6844 (0.0708) \| 1.33 (0.48) \| \| Total \| - \| 0.8371 (0.0842) \| 0.7285 (0.1199) \| 1.68 (1.10) \| \| Data are presented as mean (standard deviation). Abbreviations: AD, Alzheimer’s disease; MCI, mild cognitive impairment; ADNI, Alzheimer’s Disease Neuroimaging Initiative; HD95, 95th percentile Hausdorff distance. \| \| \| \| \|  \| **Table S2 Segmentation Accuracy of 3D UX-Net Compared to Manually Refined Masks** \| \| \| \| \| \| --- \| --- \| --- \| --- \| --- \| \| Group \| Subgroup \| Dice Score \| Jaccard Index \| HD95 \| \| ADNI dataset \| MCI (n=20) \| 0.9738 (0.0123) \| 0.9493 (0.0229) \| 0.47 (0.50) \| \| AD (n=20) \| 0.9624 (0.0204) \| 0.9283 (0.0369) \| 0.49 (0.56) \| \| In-House dataset \| MCI (n=5) \| 0.9785 (0.0127) \| 0.9582 (0.0242) \| 0.20 (0.40) \| \| AD (n=5) \| 0.9768 (0.0139) \| 0.9549 (0.0262) \| 0.20 (0.40) \| \| Total \| - \| 0.9702 (0.0174) \| 0.9426 (0.0319) \| 0.43 (0.52) \| \| Data are presented as mean (standard deviation). Abbreviations: AD, Alzheimer’s disease; MCI, mild cognitive impairment; ADNI, Alzheimer’s Disease Neuroimaging Initiative; HD95, 95th percentile Hausdorff distance. \| \| \| \| \|  \| **Table S3 Performance metrics of radiomics feature-based classification models** \| \| \| \| \| \| \| --- \| --- \| --- \| --- \| --- \| --- \| \| **Model** \| **Classifier** \| **AUC (95% CI)** \| **Precision** \| **Recall** \| **Specificity** \| \| AD vs MCI \| SVM \| 0.794 (0.741 - 0.847) \| 0.727 \| 0.688 \| 0.768 \| \| RF \| 0.771 (0.711 - 0.827) \| 0.710 \| 0.688 \| 0.746 \| \| XGBoost \| 0.748 (0.686 - 0.806) \| 0.725 \| 0.578 \| 0.803 \| \| LightGBM \| 0.740 (0.676 - 0.802) \| 0.734 \| 0.625 \| 0.796 \| \| RC \| 0.785 (0.732 - 0.839) \| 0.719 \| 0.680 \| 0.761 \| \| NB \| 0.754 (0.692 - 0.808) \| 0.733 \| 0.602 \| 0.803 \| \| LDA \| 0.791 (0.739 - 0.846) \| 0.699 \| 0.742 \| 0.711 \| \| QDA \| 0.773 (0.718 - 0.829) \| 0.726 \| 0.664 \| 0.775 \| \| GBM \| 0.732 (0.668 - 0.795) \| 0.743 \| 0.609 \| 0.810 \| \| Enet \| 0.792(0.738 - 0.846) \| 0.696 \| 0.750 \| 0.704 \| \| plsRglm \| 0.783 (0.730 - 0.838) \| 0.719 \| 0.680 \| 0.761 \| \| LASSO \| 0.790 (0.736 - 0.844) \| 0.702 \| 0.719 \| 0.725 \| \|  \|  \|  \|  \|  \|  \| \| MCI-s vs MCI-p \| SVM \| 0.709 (0.505 - 0.891) \| 0.688 \| 0.786 \| 0.643 \| \| RF \| 0.686 (0.474 - 0.882) \| 0.647 \| 0.786 \| 0.571 \| \| XGBoost \| 0.714 (0.500 - 0.887) \| 0.889 \| 0.571 \| 0.929 \| \| LightGBM \| 0.709 (0.500 - 0.891) \| 0.778 \| 0.500 \| 0.857 \| \| RC \| 0.740 (0.550 - 0.908) \| 0.684 \| 0.929 \| 0.571 \| \| NB \| 0.653 (0.446 - 0.856) \| 0.667 \| 0.857 \| 0.571 \| \| LDA \| 0.714 (0.505 - 0.896) \| 0.684 \| 0.929 \| 0.571 \| \| QDA \| 0.730 (0.524 - 0.898) \| 0.650 \| 0.929 \| 0.500 \| \| GBM \| 0.656 (0.433 - 0.844) \| 0.625 \| 0.714 \| 0.571 \| \| Enet \| 0.730 (0.535 - 0.901) \| 0.684 \| 0.928 \| 0.571 \| \| plsRglm \| 0.745 (0.547 - 0.911) \| 0.687 \| 0.920 \| 0.571 \| \| LASSO \| 0.740 (0.550 - 0.908) \| 0.684 \| 0.929 \| 0.571 \|   Abbreviations: MCI, mild cognitive impairment; AD, Alzheimer's disease; MCI-s, MCI-stable; MCI-p, MCI-progression; SVM, Support Vector Machine; RF, Random Forest; XGBoost, eXtreme Gradient Boosting; LightGBM, Light Gradient Boosting Machine; RC, Ridge Classifier; NB, Naive Bayes; LDA, Linear Discriminant Analysis; QDA, Quadratic Discriminant Analysis; GBM, Gradient Boosting Machine; Enet, Elastic Net; plsRglm, Partial Least Squares Regression Generalized Linear Model; LASSO, Least Absolute Shrinkage and Selection Operator, AUC, area under the curve; 95% CI, 95% confidence interval.   \| **Table S4 Performance metrics of classification models in the external validation cohort** \| \| \| \| \| \| \| --- \| --- \| --- \| --- \| --- \| --- \| \| **Model** \| **Classifier** \| **AUC (95% CI)** \| **Precision** \| **Recall** \| **Specificity** \| \| AD vs MCI \| SVM \| 0.657 (0.442 - 0.848) \| 0.565 \| 0.929 \| 0.333 \| \| RF \| 0.750 (0.543 - 0.900) \| 0.692 \| 0.643 \| 0.733 \| \| XGBoost \| 0.786 (0.586 - 0.929) \| 0.769 \| 0.714 \| 0.800 \| \| LightGBM \| 0.779 (0.592 - 0.924) \| 0.714 \| 0.714 \| 0.733 \| \| RC \| 0.657 (0.426 - 0.838) \| 0.609 \| 1.000 \| 0.400 \| \| NB \| 0.676 (0.456 - 0.848) \| 0.583 \| 1.000 \| 0.333 \| \| LDA \| 0.667 (0.438 - 0.849) \| 0.609 \| 1.000 \| 0.400 \| \| QDA \| 0.657 (0.433 - 0.853) \| 0.857 \| 0.429 \| 0.933 \| \| GBM \| 0.538 (0.321 - 0.743) \| 0.562 \| 0.643 \| 0.533 \| \| Enet \| 0.500 (0.500 - 0.500) \| 0.000 \| 0.000 \| 1.000 \| \| plsRglm \| 0.695 (0.481 - 0.870) \| 0.625 \| 0.714 \| 0.600 \| \| LASSO \| 0.500 (0.500 - 0.500) \| 0.000 \| 0.000 \| 1.000 \| \| Abbreviations: MCI, mild cognitive impairment; AD, Alzheimer's disease; SVM, Support Vector Machine; RF, Random Forest; XGBoost, eXtreme Gradient Boosting; LightGBM, Light Gradient Boosting Machine; RC, Ridge Classifier; NB, Naive Bayes; LDA, Linear Discriminant Analysis; QDA, Quadratic Discriminant Analysis; GBM, Gradient Boosting Machine; Enet, Elastic Net; plsRglm, Partial Least Squares Regression Generalized Linear Model; LASSO, Least Absolute Shrinkage and Selection Operator, AUC, area under the curve; 95% CI, 95% confidence interval. \| \| \| \| \| \| \|  \| \| \| \| \| \| \| \| \| \| \| \| \| \| \| \| \| --- \| --- \| --- \| --- \| --- \| --- \| --- \| --- \| --- \| --- \| --- \| --- \| --- \| --- \| --- \| --- \| --- \| --- \| --- \| --- \| --- \| --- \| --- \| --- \| --- \| --- \| --- \| --- \| --- \| --- \| --- \| --- \| --- \| --- \| --- \| --- \| --- \| --- \| --- \| --- \| --- \| --- \| --- \| --- \| --- \| --- \| --- \| --- \| --- \| --- \| --- \| --- \| --- \| --- \| --- \| --- \| --- \| --- \| --- \| --- \| --- \| --- \| --- \| --- \| --- \| --- \| --- \| --- \| --- \| --- \| --- \| --- \| --- \| --- \| --- \| --- \| --- \| --- \| --- \| --- \| --- \| --- \| --- \| --- \| --- \| --- \| --- \| --- \| --- \| --- \| --- \| --- \| --- \| --- \| --- \| --- \| --- \| --- \| --- \| --- \| --- \| --- \| --- \| --- \| --- \| --- \| --- \| --- \| --- \| --- \| --- \| --- \| --- \| --- \| --- \| --- \| --- \| --- \| --- \| --- \| --- \| --- \| --- \| --- \| --- \| --- \| --- \| --- \| --- \| --- \| --- \| --- \| --- \| --- \| --- \| --- \| --- \| --- \| --- \| --- \| --- \| --- \| --- \| --- \| --- \| --- \| --- \| --- \| --- \| --- \| --- \| --- \| --- \| --- \| --- \| --- \| --- \| --- \| --- \| --- \| --- \| --- \| --- \| --- \| --- \| --- \| --- \| --- \| --- \| --- \| --- \| --- \| --- \| --- \| --- \| --- \| --- \| --- \| --- \| --- \| --- \| --- \| --- \| --- \| --- \| --- \| --- \| --- \| --- \| --- \| --- \| --- \| --- \| --- \| --- \| --- \| --- \| --- \| --- \| --- \| --- \| --- \| --- \| --- \| --- \| --- \| --- \| --- \| --- \| --- \| --- \| --- \| --- \| --- \| --- \| --- \| --- \| --- \| --- \| --- \| --- \| --- \| --- \| --- \| --- \| --- \| --- \| --- \| --- \| --- \| --- \| --- \| --- \| --- \| --- \| --- \| --- \| --- \| --- \| --- \| --- \| --- \| --- \| --- \| --- \| --- \| --- \| --- \| --- \| --- \| --- \| --- \| --- \| --- \| --- \| --- \| --- \| --- \| --- \| --- \| --- \| --- \| --- \| --- \| --- \| --- \| --- \| --- \| --- \| --- \| --- \| --- \| --- \| --- \| --- \| --- \| --- \| --- \| --- \| --- \| --- \| --- \| --- \| --- \| --- \| --- \| --- \| --- \| --- \| --- \| --- \| --- \| --- \| --- \| --- \| --- \| --- \| --- \| --- \| --- \| --- \| --- \| --- \| --- \| --- \| --- \| --- \| --- \| --- \| --- \| --- \| \| **Table S5 Performance metrics of clinical and radiomics feature-based classification models** \| \| \| \| \| \| \| \| \| \| \| **Model** \| \| **Classifier** \| \| **AUC (95% CI)** \| \| **Precision** \| **Recall** \| \| **Specificity** \| \| AD vs MCI \| \| SVM \| \| 0.907 (0.848 - 0.953) \| \| 0.885 \| 0.754 \| \| 0.885 \| \| RF \| \| 0.865 (0.791 - 0.927) \| \| 0.857 \| 0.787 \| \| 0.846 \| \| XGBoost \| \| 0.892 (0.825- 0.944) \| \| 0.864 \| 0.836 \| \| 0.846 \| \| LightGBM \| \| 0.906 (0.841 - 0.955) \| \| 0.895 \| 0.836 \| \| 0.885 \| \| RC \| \| 0.901 (0.843 - 0.948) \| \| 0.915 \| 0.705 \| \| 0.923 \| \| NB \| \| 0.863 (0.791 - 0.923) \| \| 0.950 \| 0.623 \| \| 0.962 \| \| LDA \| \| 0.892 (0.830 - 0.942) \| \| 0.898 \| 0.721 \| \| 0.904 \| \| QDA \| \| 0.906 (0.848 - 0.949) \| \| 0.860 \| 0.803 \| \| 0.846 \| \| GBM \| \| 0.863 (0.789 - 0.923) \| \| 0.915 \| 0.705 \| \| 0.923 \| \| Enet \| \| 0.895(0.834 - 0.944) \| \| 0.868 \| 0.754 \| \| 0.865 \| \| plsRglm \| \| 0.896 (0.837 - 0.945) \| \| 0.974 \| 0.623 \| \| 0.981 \| \| LASSO \| \| 0.894 (0.834 - 0.942) \| \| 0.898 \| 0.721 \| \| 0.904 \| \|  \| \|  \| \|  \| \|  \|  \| \|  \| \| MCI-s vs MCI-p \| \| SVM \| \| 0.900 (0.733 - 1.000) \| \| 0.769 \| 1.000 \| \| 0.750 \| \| RF \| \| 0.875 (0.692 - 1.000) \| \| 0.889 \| 0.800 \| \| 0.917 \| \| XGBoost \| \| 0.792 (0.596 - 0.967) \| \| 0.778 \| 0.700 \| \| 0.833 \| \| LightGBM \| \| 0.825 (0.625 - 0.967) \| \| 0.727 \| 0.800 \| \| 0.750 \| \| RC \| \| 0.908 (0.744 - 1.000) \| \| 0.833 \| 1.000 \| \| 0.833 \| \| NB \| \| 0.833 (0.632 - 0.983) \| \| 0.750 \| 0.900 \| \| 0.750 \| \| LDA \| \| 0.900 (0.744 - 1.000) \| \| 0.818 \| 0.900 \| \| 0.833 \| \| QDA \| \| 0.900 (0.744 - 1.000) \| \| 0.818 \| 0.900 \| \| 0.833 \| \|  \| GBM \| \| \| 0.833 (0.641 - 0.975) \| \| 0.667 \| 1.000 \| \| 0.583 \| \| Enet \| \| \| 0.833 (0.696 - 1.000) \| \| 0.900 \| 0.900 \| \| 0.917 \| \|  \| plsRglm \| \| \| 0.900 (0.744 - 1.000) \| \| 0.818 \| 0.900 \| \| 0.833 \| \| LASSO \| \| \| 0.875 (0.678 - 1.000) \| \| 0.900 \| 0.900 \| \| 0.917 \| \| Abbreviations: MCI, mild cognitive impairment; AD, Alzheimer's disease; MCI-s, MCI-stable; MCI-p, MCI-progression; SVM, Support Vector Machine; RF, Random Forest; XGBoost, eXtreme Gradient Boosting; LightGBM, Light Gradient Boosting Machine; RC, Ridge Classifier; NB, Naive Bayes; LDA, Linear Discriminant Analysis; QDA, Quadratic Discriminant Analysis; GBM, Gradient Boosting Machine; Enet, Elastic Net; plsRglm, Partial Least Squares Regression Generalized Linear Model; LASSO, Least Absolute Shrinkage and Selection Operator. AUC, area under the curve; 95% CI, 95% confidence interval. \| \| \| \| \| \| \| \| \| \| \| **Table S6 Performance metrics of clinical feature-based classification models** \| \| \| \| \| \| \| \| \| \| \| **Model** \| **Classifier** \| \| **AUC (95% CI)** \| \| **Precision** \| \| \| **Recall** \| **Specificity** \| \| AD vs MCI \| SVM \| \| 0.770 (0.697 - 0.839) \| \| 0.689 \| \| \| 0.785 \| 0.696 \| \| RF \| \| 0.738 (0.658 - 0.814) \| \| 0.631 \| \| \| 0.886 \| 0.554 \| \| XGBoost \| \| 0.744 (0.669 - 0.819) \| \| 0.685 \| \| \| 0.772 \| 0.696 \| \| LightGBM \| \| 0.788 (0.718 - 0.855) \| \| 0.747 \| \| \| 0.747 \| 0.783 \| \| RC \| \| 0.804 (0.731 - 0.868) \| \| 0.720 \| \| \| 0.848 \| 0.717 \| \| NB \| \| 0.749 (0.671 - 0.823) \| \| 0.631 \| \| \| 0.886 \| 0.554 \| \| LDA \| \| 0.779 (0.707 - 0.850) \| \| 0.716 \| \| \| 0.797 \| 0.728 \| \| QDA \| \| 0.756 (0.679 - 0.828) \| \| 0.674 \| \| \| 0.785 \| 0.674 \| \| GBM \| \| 0.737 (0.660 - 0.811) \| \| 0.644 \| \| \| 0.848 \| 0.598 \| \| Enet \| \| 0.782 (0.708 - 0.852) \| \| 0.713 \| \| \| 0.848 \| 0.707 \| \| plsRglm \| \| 0.809 (0.739 - 0.872) \| \| 0.727 \| \| \| 0.810 \| 0.739 \| \| LASSO \| \| 0.784 (0.710 - 0.853) \| \| 0.686 \| \| \| 0.911 \| 0.641 \| \|  \|  \| \|  \| \|  \| \| \|  \|  \| \| MCI-s vs MCI-p \| SVM \| \| 0.663 (0.432 - 0.862) \| \| 0.714 \| \| \| 0.714 \| 0.714 \| \| RF \| \| 0.776 (0.592 - 0.931) \| \| 0.684 \| \| \| 0.929 \| 0.571 \| \| XGBoost \| \| 0.714 (0.510 - 0.897) \| \| 0.714 \| \| \| 0.714 \| 0.714 \| \| LightGBM \| \| 0.673 (0.449 - 0.893) \| \| 0.818 \| \| \| 0.643 \| 0.857 \| \| RC \| \| 0.653 (0.438 - 0.850) \| \| 0.643 \| \| \| 0.643 \| 0.643 \| \| NB \| \| 0.597 (0.379 - 0.818) \| \| 0.700 \| \| \| 0.500 \| 0.786 \| \| LDA \| \| 0.643 (0.428 - 0.844) \| \| 0.643 \| \| \| 0.643 \| 0.643 \| \| QDA \| \| 0.673 (0.468 - 0.861) \| \| 0.778 \| \| \| 0.500 \| 0.857 \| \| GBM \| \| 0.742 (0.556 - 0.918) \| \| 0.706 \| \| \| 0.857 \| 0.643 \| \| Enet \| \| 0.617 (0.396 - 0.844) \| \| 0.692 \| \| \| 0.643 \| 0.714 \| \| plsRglm \| \| 0.673 (0.468 - 0.861) \| \| 0.778 \| \| \| 0.500 \| 0.857 \| \| LASSO \| \| 0.607 (0.383 - 0.836) \| \| 0.692 \| \| \| 0.643 \| 0.714 \|   Abbreviations: MCI, mild cognitive impairment; AD, Alzheimer's disease; MCI-s, MCI-stable; MCI-p, MCI-progression; SVM, Support Vector Machine; RF, Random Forest; XGBoost, eXtreme Gradient Boosting; LightGBM, Light Gradient Boosting Machine; RC, Ridge Classifier; NB, Naive Bayes; LDA, Linear Discriminant Analysis; QDA, Quadratic Discriminant Analysis; GBM, Gradient Boosting Machine; Enet, Elastic Net; plsRglm, Partial Least Squares Regression Generalized Linear Model; LASSO, Least Absolute Shrinkage and Selection Operator, AUC, area under the curve; 95% CI, 95% confidence interval.   \| **Table S7 Comparison of representative CP radiomic features across different Aβ and p-Tau pathology stages** \| \| \| \| \| \| \| --- \| --- \| --- \| --- \| --- \| --- \| \| **Feature** \| ***F*** \| ***p*** \| ***p*-value^a^** \| ***p*-value^b^** \| ***p*-value^c^** \| \| WaveletHHH_glszm_ZonePercentage \| 13.680 \| ＜0.001 \| ＜0.001 \| 0.003 \| 0.007 \| \| WaveletHHH_gldm_SmallDependenceEmphasis \| 13.017 \| ＜0.001 \| ＜0.001 \| 0.005 \| 0.005 \| \| WaveletHHH_gldm_DependenceNonUniformityNormalized \| 12.310 \| ＜0.001 \| ＜0.001 \| 0.004 \| 0.012 \| \| WaveletHHH_glrlm_RunVariance \| 12.020 \| ＜0.001 \| ＜0.001 \| 0.002 \| 0.023 \| \| WaveletHHH_glrlm_RunPercentage \| 12.017 \| ＜0.001 \| ＜0.001 \| 0.003 \| 0.018 \| \| WaveletHHH_glrlm_RunLengthNonUniformityNormalized \| 12.004 \| ＜0.001 \| ＜0.001 \| 0.003 \| 0.016 \| \| Gradient_gldm_DependenceNonUniformityNormalized \| 11.955 \| ＜0.001 \| ＜0.001 \| 0.001 \| 0.038 \| \| WaveletHHH_glrlm_LongRunEmphasis \| 11.861 \| ＜0.001 \| ＜0.001 \| 0.003 \| 0.021 \| \| WaveletHHH_glrlm_ShortRunEmphasis \| 11.850 \| ＜0.001 \| ＜0.001 \| 0.003 \| 0.018 \| \| Gradient_glszm_ZonePercentage \| 11.546 \| ＜0.001 \| ＜0.001 \| 0.001 \| 0.049 \| \| WaveletHHH_gldm_LargeDependenceEmphasis \| 11.122 \| ＜0.001 \| ＜0.001 \| 0.003 \| 0.028 \| \| Gradient_ngtdm_Contrast \| 10.998 \| ＜0.001 \| ＜0.001 \| 0.003 \| 0.033 \| \| WaveletHHH_gldm_DependenceVariance \| 10.244 \| ＜0.001 \| ＜0.001 \| 0.004 \| 0.040 \| \| WaveletHHH_glcm_Id \| 10.151 \| ＜0.001 \| ＜0.001 \| 0.004 \| 0.044 \| \| WaveletHHH_glcm_Idm \| 10.115 \| ＜0.001 \| ＜0.001 \| 0.004 \| 0.042 \| \| Exponential_glcm_DifferenceVariance \| 9.816 \| ＜0.001 \| ＜0.001 \| 0.034 \| 0.007 \| \| Exponential_glcm_Contrast \| 9.787 \| ＜0.001 \| ＜0.001 \| 0.042 \| 0.005 \| \| Exponential_glcm_Idmn \| 9.744 \| ＜0.001 \| ＜0.001 \| 0.048 \| 0.005 \| \| Gradient_glcm_DifferenceVariance \| 9.678 \| ＜0.001 \| ＜0.001 \| 0.008 \| 0.031 \| \| Exponential_ngtdm_Complexity \| 8.916 \| ＜0.001 \| ＜0.001 \| 0.042 \| 0.010 \| \| Logarithm_gldm_SmallDependenceHighGrayLevelEmphasis \| 8.799 \| ＜0.001 \| ＜0.001 \| 0.021 \| 0.022 \| \| Logarithm_glszm_SmallAreaHighGrayLevelEmphasis \| 8.387 \| ＜0.001 \| ＜0.001 \| 0.023 \| 0.027 \| \| WaveletHHH_glszm_LargeAreaLowGrayLevelEmphasis \| 8.118 \| ＜0.001 \| ＜0.001 \| 0.017 \| 0.043 \| \| WaveletHHH_gldm_LargeDependenceLowGrayLevelEmphasis \| 8.095 \| ＜0.001 \| ＜0.001 \| 0.017 \| 0.045 \| \| Square_glcm_DifferenceVariance \| 8.082 \| ＜0.001 \| ＜0.001 \| 0.019 \| 0.040 \| \| Squareroot_gldm_SmallDependenceHighGrayLevelEmphasis \| 8.042 \| ＜0.001 \| ＜0.001 \| 0.031 \| 0.025 \| \| WaveletHHH_ngtdm_Contrast \| 7.991 \| ＜0.001 \| ＜0.001 \| 0.032 \| 0.026 \| \| Squareroot_glszm_SmallAreaHighGrayLevelEmphasis \| 7.827 \| ＜0.001 \| ＜0.001 \| 0.032 \| 0.029 \| \| Square_glcm_Contrast \| 7.506 \| 0.001 \| ＜0.001 \| 0.023 \| 0.048 \| \| Square_glcm_Idmn \| 7.396 \| 0.001 \| ＜0.001 \| 0.025 \| 0.049 \| \| WaveletHHH_gldm_SmallDependenceHighGrayLevelEmphasis \| 6.821 \| 0.001 \| ＜0.001 \| 0.045 \| 0.041 \| \| The *F*-value and its corresponding *p* were derived from the one-way ANOVA F-test comparing the three groups.  *p*-value^a^: Aβ+＆p-Tau+ vs. Aβ+＆p-Tau-/Aβ-＆p-Tau+;  *p*-value^b^: Aβ+＆p-Tau-/Aβ-＆p-Tau+ vs. Aβ-＆p-Tau-;  *p*-value^c^: Aβ+＆p-Tau+ vs. Aβ-＆p-Tau-. \| \| \| \| \| \| |  |
